# Towards Digital Twin-Oriented Complex Networked Systems: Introducing heterogeneous node features and interaction rules

**DOI:** 10.1371/journal.pone.0296426

**Published:** 2024-01-02

**Authors:** Jiaqi Wen, Bogdan Gabrys, Katarzyna Musial

**Affiliations:** Complex Adaptive Systems, Data Science Institute, University of Technology Sydney, Sydney, NSW, Australia; LUMSA: Libera Universita Maria Santissima Assunta, ITALY

## Abstract

This study proposes an extendable modelling framework for Digital Twin-Oriented Complex Networked Systems (DT-CNSs) with a goal of generating networks that faithfully represent real-world social networked systems. Modelling process focuses on (i) features of nodes and (ii) interaction rules for creating connections that are built based on individual node’s preferences. We conduct experiments on simulation-based DT-CNSs that incorporate various features and rules about network growth and different transmissibilities related to an epidemic spread on these networks. We present a case study on disaster resilience of social networks given an epidemic outbreak by investigating the infection occurrence within specific time and social distance. The experimental results show how different levels of the structural and dynamics complexities, concerned with feature diversity and flexibility of interaction rules respectively, influence network growth and epidemic spread. The analysis revealed that, to achieve maximum disaster resilience, mitigation policies should be targeted at nodes with preferred features as they have higher infection risks and should be the focus of the epidemic control.

## Introduction

Over the years, Complex Networked Systems (CNSs) have been modelled with increasing structural complexity levels that resulted in models with higher and higher accuracy. The ultimate goal of these research efforts is to build models that are an accurate reflection and, sometimes, extension of reality. Family of models that perfectly reflect real world networked systems can be seen as Digital Twin Oriented Complex Networked Systems (DT-CNSs) [1].

In DT-CNS space, Digital Twins (DTs) are expected to provide an accurate reflection and extension of reality, which enables us to recreate existing scenarios and conduct what-if analyses on scenarios that have never happened before. A DT is an ultimate goal, rather than a modelling approach or paradigm, for the representation and modelling of Complex Networked Systems, which has not been realised yet. The modelling and extension of CNS in the context of DT are to represent the observable information faithfully in the form of a network together with dynamics of and on this network, while enabling the fulfilment of the model’s aim and, in its ultimate realisation, seamlessly intertwining and interacting with reality in real-time. The DT-CNSs of social networked systems, as they approach a DT, focus on temporal spreading processes on temporal social networks, characterised by continuous, interrelated changes and a real-time feedback loop that enables the two-way real-time information acquisition between the DT-CNS and reality [1]. There are no current realisations of these conceptual ideas in social network space and the goal of the research presented in this paper is to build and asses an extendable DT-CNS for social networked systems that can bring us closer to the ultimate goal of DT.

The complexity of CNSs results from the heterogeneity of CNSs’ components and interactions between them as well as dynamic processes (e.g. epidemic spread) on those systems [1]. For example, the CNS, which models a social networked system, is composed of (i) the social networks concerning the nodes (e.g. people), edges (e.g. interactions, relationships, etc.) and their corresponding attributes (which describe the features of nodes and edges), and (ii) dynamic processes that aim to model the spreading phenomena on social networks, such as epidemic spread [2], opinion spread [3], rumour spread [4], news spread [5], etc.

Current studies on CNSs focus on the least complex scenario where a single dynamic process takes place on a static network without changes of the network components (nodes and edges) or the process parameters [6–11]. In addition, the spreading phenomenon on the networks is generally modelled by classic epidemiologic models such as the Susceptible-Infected (SI) model and its extensions, including the Susceptible-Infected-Susceptible (SIS) model [12], Susceptible-Infected-Recovered (SIR) model [13] and the Susceptible-Exposed-Infectious-Recovered (SEIR) model [14]. Therefore, to increase complexity of modelling, and in this way to approach Digital Twin, a natural way forward is to increase the heterogeneity of nodes’ features (characteristics of each specific node) and their preferences to create relationships as well as allow the system to evolve over time, both from the perspective of structure and process. To enable the flexible extension of a DT-CNS and its generic applications across disciplines, the Susceptible-Infected (SI) model can serve as a promising starting point to model the spreading phenomenon on the networks with increasing complexity.

The complexity of modelling CNS is constrained by the system’s observability. The observability is concerned with the ability to faithfully reconstruct the state of a system from a limited set of measured variables in finite time [1, 15]. It not only determines the available ground-truth information to be represented and modelled but also the information to be simulated towards achieving the ultimate goal of a Digital Twin (DT). With different levels of observability, we categorise the CNS components as data-driven, simulation-based and hybrid [1], each composed of real data [16–18], purely simulated data [19] and both real and simulated data respectively. Current studies generally build CNSs with hybrid components (e.g. real networks and simulated dynamic processes) due to the partial observability of a real-world scenario [5–11, 20, 21].

To achieve a faithful CNS representation of a real-world social networked system, we need to preserve as much information as needed in the modelling process for a better model performance under the observability constraints [1]. The evaluation of model performance in mimicking a social networked system generally involves the discussion on the similarity of approaching complex real-world patterns [19, 22, 23], the simulation efficiency [1, 24] and the ability to reproduce the fundamental results with the purpose of result verification and model extension [25]. However, there is a need to clarify the evaluation protocol on faithfulness considering the requirements on CNS model performance and their relations with the observability constraints, which thus poses a research gap to be addressed in this study.

In this study, we propose an extendable modelling framework for the DT-CNSs of social networked system, composed of (i) a flexible social network model that allows for static network formation based on heterogeneous node features and interaction rules related to connection preferences, and (ii) a epidemic spreading process model for the Susceptible-Infected (SI) spread with a predetermined seed selection strategy and an infection rate. We propose a set of evaluation metrics to validate the faithfulness of DT-CNSs, considering their similarity with real system, efficiency and reproducibility of the network patterns and the infection occurrence on such networks. We present a case study on the disaster resilience of social networks considering their infection occurrence within specific time and social distance given an epidemic outbreak. Our experiments focus on various simulated networks, where nodes have heterogeneous attributes and the interactions between the nodes are driven by various rules (e.g. social networks driven by age features and an interaction rule—preference for a similar age when nodes interact). The results show that the heterogeneous features and interaction rules (related to preference for features) influence the network structure and can enhance epidemic outbreak. Nodes with preferred by others features have more connections and, as a result, get exposed to higher infection risk within the same proximity to the initial infection node. Finally, we have implemented an open source python toolbox with all the functionalities of the proposed modelling framework and facilitating its extension towards a DT-CNS with higher complexity levels (https://github.com/UTS-CASLab/DT-CNS).

The main contributions of this study are:

- Constructing an extendable DT-CNS modelling framework composed of a network model based on heterogeneous nodes’ features and rules driving the interactions between nodes and a process model spreading over the network;
- Creating an evaluation protocol for faithfulness, concerned with similarity, efficiency and reproducibility;
- Validating the influence of heterogeneous nodes’ features and interaction rules on network growth and level of epidemic spread;
- Suggesting disaster mitigation policies dependent on age diversity and social preference.

The rest of this study is structured as follows. Background provides background in the space of dynamics of and on the networks. Digital Twin Orientated Complex Networked System presents the methodology of building and driving a CNS towards a DT. Following this, Results builds and evaluates the simulation-based CNSs. Finally, in Conclusion, we conclude the study and outline future directions.

## Background

This section presents the network and process models developed and employed in the current studies in Network and process models. It discusses (i) interaction rules that drive the network growth through preference for node features and (ii) transmission rules governing the node adaptability to an epidemic spread in different conditions. We also discuss the evaluation metrics involved in DT-CNS research in Evaluation metrics, including (i) complexity of DT-CNSs, (ii) performance in mimicking real-world social networked systems and (ii) faithfulness that pose different requirements in.

### Network and process models

#### Network model

Network model aims at providing a faithful representation of a network that minimises information loss [1]. There are two widely employed principles for network formation and growth: preferential attachment [26–29] and homophily [30–33]. By incorporating these two principles, this study starts to build DT-CNSs based on observable networked information.

**Preferential attachment** describes the “rich get richer” principle [27], where highly connected nodes increase their connectivity faster than their less-connected peers [26]. A scale-free network model based on the preferential attachment to higher node degrees was first proposed in [28]. With time, more and more studies in the network science space started looking into other topological features, such as clustering coefficient, closeness centrality and eigenvector centrality [27, 29]. In our study, we allow preferential attachment to the nodes with specific feature values concerned with topology and attributes.

**Homophily** desribes the tendency to associate ourselves with similar others [30]. It can either be choice homophily or induced homophily. The first one is a preference for similar attributes, e.g. people tend to interact with those similar interests. Induced homophily is a result of the interaction opportunities due to structural proximity [31–33] (e.g. people who interact because they work in the same building). In the network embedding paradigms, the induced homophily generally suggests that highly connected nodes should be embedded closely in the latent representation space [34]. In contrast, the choice homophily effect leads to the consideration of attribute proximity [35]. In our study, we account for both choice homophily and induced homophily based on the proximity of structures and attributes.

#### Process model

Process model for the spreading phenomenon on networks has two components: (i) seed selection strategy for the first contagious nodes, and (ii) the transmissibility that describes the spread conditional on node features [36–38].

**Seed selection** strategies identify single/multiple seeds (source nodes of the spreading process) in the initial/subsequent steps (single-stage seeding/sequential seeding) from which the spread starts. Current studies generally select seeds based on the centrality measures, such as degree centrality, betweenness centrality, closeness centrality and eigenvector centrality [39, 40] or identify driver nodes and using them as seeds [38].

**Transmissibility** involved in the contagion models depends on many, different elements. For example, node adoptability, concerning the probability to adopt a spread, rises when there are multiple exposures for multiple contagions/simplicial contagion, compared with a simple contagion [20, 41]. In addition, the transmissibility can also vary with specific interactions (e.g. physical contact, airborne spread, etc.) among node pairs or groups [42]. Particularly, [41] explains the current contagion models with conditional transmissibility, such as the threshold model, stochastic contagion model, diffusion percolation model, independent cascade model and Reed–Frost model.

Overall, current network models are built on interaction rules, either related to preference for topological features (preferential attachment) or preference for similar node attributes (homophily). Current rule-based process models focus on seed selection strategies and transmission rules. However, none of the studies combines heterogeneous features and interaction rules and investigates their influence on the network growth and the corresponding epidemic spread. Therefore, this study proposes an extendable modelling framework for DT-CNSs based on heterogeneous node features and diverse interaction rules.

### Evaluation metrics

#### Complexity

Complexity metrics, in DT-CNS space, focus on the conceptual differences in DT-CNSs and the quantitative patterns generated by DT-CNSs.

**Complexity generations**, proposed in our previous survey on CNSs conceptually evaluate the complexity of DT-CNSs and guide their extension towards a DT, which progress from the generation 1 DT-CNS to the generation 5 DT-CNS (a DT) with an increasing complexity level [1]. The DT-CNSs, in different complexity generations, each systemically vary in three key aspects: evolvability in dynamics of networks and processes, interrelations in these dynamics and their interplay with the real world.

**Fig 1:**
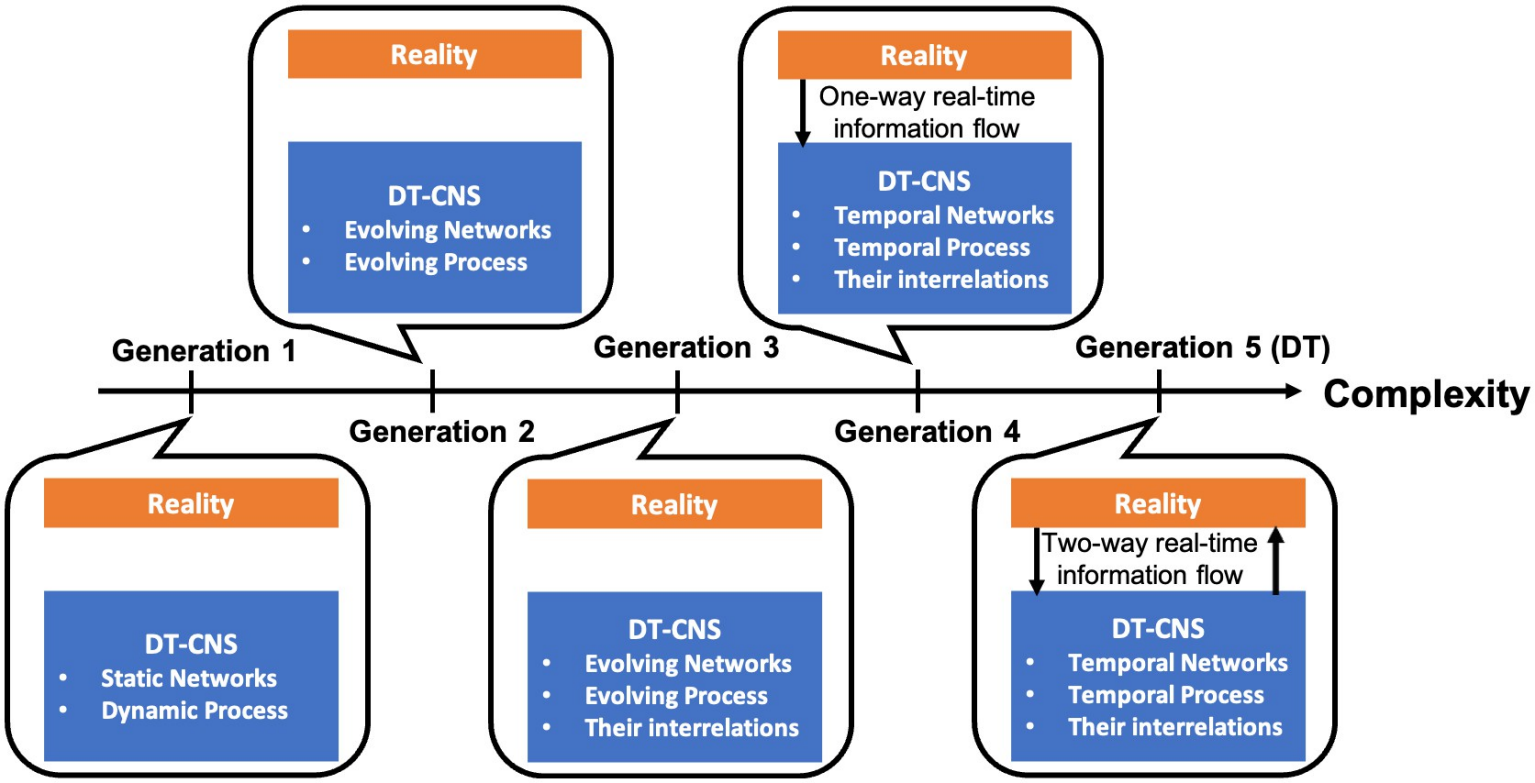
The generations of DT-CNSs that progress with increasing complexity levels towards a DT.

As shown in Fig 1, in generation 1, the DT-CNSs focus on dynamic processes on static networks. The generation 2 DT-CNSs focus on the evolving networks and processes, whose dynamics change over time and are captured in snapshots. In generation 3, the DT-CNS get even more complex when considering the interrelations between the evolving dynamics in DT-CNSs. The models have a paradigm shift in generation 4 as DT-CNSs focus on instantaneous changes in DT-CNS and model temporal dynamic processes on temporal networks with interrelations between them and the continuous acquisition of real-time information. The generation 5 DT-CNSs further introduce the generation 4 DT-CNSs with a feedback loop between the CNSs and the real system, approaching the ultimate goal of a DT. More specifically, the generation 5 DT-CNSs (DTs) focus on temporal dynamic processes on temporal networks with interrelations between them and a real-time feedback loop that enables the two-way real-time information flow and acquisition between the DT-CNS and reality [1]. For example, in the generation 5 DT-CNSs (DT) of the social networked systems, the real-time interrelated changes of epidemic spread and social interactions can be observed and modelled simultaneously, which simulation results for the unforeseeable future can be fed back to reality to assist policy making.

From generation 1 to generation 5, the real system can be represented more faithfully with richer information captured, and finally a CNS-based DT can be created in generation 5 [1]. Current studies generally fall in generation 1 and generation 2. This study aims to build an extendable DT-CNS framework in generation 1 that can be further extended towards higher complexity generations and approach a DT.

**Complexity measures** quantitatively evaluate the complex patterns emerging from the DT-CNSs. In a DT-CNS of the social networked systems in an epidemic outbreak, we focus on the network patterns concerning the network topology and attributes, and the epidemic spreading patterns including the transmissibility of epidemic spread, and the resulting infections.

Current studies on network simulations and network analysis generally focus on the evaluation of network topology, which involves discussing the global level structure measures such as degree distribution [43], shortest path length [43] and modularity [43, 44] as well as the local structure measures such as triad [45], clustering coefficient [46] and closure coefficient [46]. In contrast, CNS studies on specific modelling tasks, such as change point detection [47], link prediction [48] and node classification [49], tend to emphasise the pin-point level characteristics including nodes [47], edges [48] and the corresponding attributes [49]. However, none of these studies analyse the distributions of attributes (features of nodes and edges) or evaluate the diversity of these attributes. Considerable studies in ecological space investigate the impact of population diversity levels on the population evolution phenomenon [50–52], which involves diversity measures such as Hill numbers [51]. There is also a need to map these measures into the research on DT-CNSs to better understand the complex attributes (features) in networks.

In addition, considerable studies investigate the epidemic spreading patterns in social networks, which involves discussing the infection numbers under the impact of different epidemic transmissibilities and social network structures [53]. The investigation of the relations between infection numbers and complex network patterns has been investigated over the years [2, 11, 54]. However, the combined quantitative evaluation of infection numbers and different complex network patterns still remains a research gap to be addressed for our future study.

#### Performance

Performance of modelling DT-CNSs, given the evaluation of the complex patterns generated by DT-CNS, can be evaluated based on DT-CNS patterns’ reproducibility, the corresponding similarity between the DT-CNS patterns with a target state (e.g. the target network topology required in link prediction tasks; etc.) as well as efficiency.

**Similarity** of the CNS representation with the target patterns observed in real-world CNSs has been evaluated and pursued as an optimisation objective over the years in considerable studies [19, 22, 23]. The target patterns, as measured by the complexity measures, range from (i) pinpoint complex patterns related to nodes and edges such as network topology and attributes and infection number [49, 55], (ii) local interaction structures which can be measured by local complexity meaures like clustering coefficient distribution [45, 56], to (iii) global level complexity metrics such as the degree distribution [24]. The similarity measures depends on the value types of these target patterns, including the values (measured by e.g. precision [48, 57]), data sequences (measured by e.g. Euclidean distances [58]) and data distributions (measured by e.g. Jensen–Shannon divergence [59]).

**Efficiency** of CNS representation and modelling has been recently discussed as a fundamental element of CNS evaluation protocol [1, 24]. This involves the efficiency of data processing, concerned with the time delays compared with the real-time data flow [60], as well as the efficiency of modelling, connected to the runtime of CNS simulations [19, 61]. Few studies have considered the modelling efficiency and proved that the runtime of the CNS simulations can be influenced by the network components, such as the number of simulated features and the network size [19, 61]. Meanwhile, developing CNS towards DT-CNSs with increasing complexity levels poses new research challenges to real-time data processing and related efficiency evaluation.

**Reproducibility** generally refers to carrying out modelling tasks with an equivalent result to the original one, aiming for model verification and extension [25]. The equivalent results may differ in ways that are not expected to be significant to the final result. The requirements of equivalence depend on the research scenarios, ranging from reproducing the same phenomenon, the same statistics, the same data to the same bits [25]. Current studies on CNS simulations generally focus on the reproducible phenomenon and statistics, such as the switchover phenomenon induced by epidemic seeding on geometric networks [62] and the power-law degree distribution from the scale-free network simulations based on preferential attachment to popular nodes [26, 29]. In contrast, CNS studies on specific modelling tasks such as link prediction [48] and node classification [49], aim at reproduce the same data as the target CNS representation. Although fulfilled to a different extent in different studies on CNSs, reproducibility remains an unclarified evaluation criterion in DT-CNS space and thus is further discussed in this study.

Overall, to faithfully represent the complex patterns existing in real-world social networked systems, we need to build a DT-CNS at a necessary complexity level that can efficiently conduct similar simulations with the target and reproduce the complex patterns observable in reality. Therefore, considering the observability constraints, this study aims to propose an evaluation protocol for faithfulness regarding specific requirements and measures for similarity, efficiency and reproducibility.

## Digital Twin Orientated Complex Networked System

In this part, we provide a description of an extendable DT-CNS simulator for real-world social networked systems in An extendable DT-CNS framework and propose an evaluation protocol for the faithfulness of DT-CNSs in An Evaluation Protocol on Faithfulness. In Optimisation towards a Digital Twin we describe the optimisation process to obtain DT-CNSs model that faithfully represent reality and approach DT.

### An extendable DT-CNS framework

The extendable DT-CNS simulator enables to model the dynamics of and on the networks. It is devised with (i) a network model based on the interaction rules of homophily and preferential attachment, as well as (ii) a process model (transmission mechanism) that uses different seed selection strategies and transmission rules dependent on varying conditions.

#### Network and network dynamics

In this study, we focus on social networks composed of nodes (people), edges (people’s interactions) and the attributes of nodes and edges. We model the interactions between nodes with network dynamics governed by the interaction rules, including (i) the preferential attachment to nodes with preferred node features and (ii) the homophily (heterophily) effect that describes nodes’ preferences for connecting with similar others. These two interaction rules have been each researched in considerable studies, as reviewed in Background, and thus, our modelling framework first proposes combining these two to characterise the preferences for features and the preferences concerning the feature differences.

**Fig 2:**
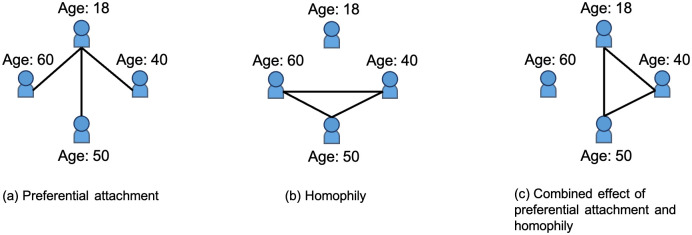
An illustrative example of network formation process based on preferential attachment (a), homophily (b), and the combination of these two interation rules (c).

We employ the following example to facilitate an understanding of the interaction rules. As shown in Fig 2, the nodes (people) in the social networks can have features such as age (e.g. age at 18, 40, 50 and 60) and evaluate the other’s age to determine their preferred connections. We assume there are only three social connections and nodes interact based on preferential attachment to young age (See Fig 2(a)), homophily effect (preferences for a similar age; See Fig 2(b)) and the combined effect of these two interaction rules (See Fig 2(c)). In Fig 2(a), the youngest node at 18 is densely connected with others due to nodes’ preferences for a young age. In Fig 2(b), the nodes of a similar age (nodes aged 40, 50 and 60) cluster due to the homophily effect. Finally, in Fig 2(c), the combination of preferential attachment and homophily effect enables the nodes aged 18, 40 and 50 to cluster based on an equally weighted preferences for young and similar ages. As we introduce more heterogeneous node features and flexible set-ups of interaction rules, the network patterns become more complex and hard to predict. Therefore, in this section, we propose the following network dynamics to incorporate the combined effect of preferential attachment and homophily and approach the real-world complex social network patterns.

Network at time *t* can be represented as *G*_*t*_ = {*V*_*t*_, *E*_*t*_, *A*_*t*_, *Z*_*t*_} based on the observable and simulated information of the changeable network components: nodes *V*_*t*_ = {*v*_1,*t*_, ⋯, *v*_*n*_*t*_, *t*_}, edges *E*_*t*_ = {*e*(*v*_*i*,*t*_, *v*_*j*,*t*_)|*v*_*i*,*t*_, *v*_*i*,*t*_ ∈ *V*, *i* ≠ *j*}, node attributes *A*_*t*_ = {*a*(*v*_1,*t*_), ⋯, *a*(*v*_*N*_*t*_, *t*_)} and the edge attributes *Z*_*t*_ = {*z*(*v*_*i*,*t*_, *v*_*j*,*t*_)|*v*_*i*,*t*_, *v*_*i*,*t*_ ∈ *V*, *i* ≠ *j*}. Network dynamics drives network formation and growth over time.

The node attribute vector *a*(*v*_*i*,*t*_) for node *v*_*i*_, referring to [19], is defined as

$$\begin{array}{c} a\left(v_{i,t}\right)=\left\{f\left(v_{i,t}\right),p\left(v_{t,i}\right),w^{p}\left(v_{t,i}\right),h\left(v_{i,t}\right),w^{h}\left(v_{t,i}\right)\right\}\;v_{i}\in V \end{array}$$ (1)

which includes an *l*-length feature vector **f**(*v*_*i*,*t*_) (*l* is the number of features) and a social-DNA (sDNA) defined with another four *l*-length vectors. Features presented in **f**(*v*_*i*,*t*_) are characteristics that describe each individual *v*_*i*,*t*_, such as age, gender and interests. **p**(*v*_*t*,*i*_) determines the preference for features with a trinary value: 0, 1, or −1, which each, respectively, indicates: 0—no preferential attachment to the corresponding node feature in **f**(*v*_*i*,*t*_), 1—preferential attachment to a higher feature value or −1—to a lower feature value. For example, the scale-free network model, where nodes connect with other popular nodes, uses pure preferential attachment (a favourable preference) to node degrees. Similarly, **h**(*v*_*t*,*i*_) determines the preference for similar features with a trinary value: 0, 1, −1, each, respectively, representing no homophily (0), heterophily (1) and homophily (−1). For example, in a social network where people like to interact with people of similar ages, we would use pure homophily to describe the negative preference for age differences. Both of these preferences are followed with a weighting factor **w**^*p*^(*v*_*t*,*i*_) and **w**^*h*^(*v*_*t*,*i*_) within the range of (0, 1].

The feature **f**(*v*_*i*,*t*_) and the sDNA {**p**(*v*_*t*,*i*_), **w**^*p*^(*v*_*t*,*i*_), **h**(*v*_*i*,*t*_), **w**^*h*^(*v*_*t*,*i*_)} can vary with nodes in a frozen time scale and/or mutate over time. They can also co-evolve with the network topology based on the rules of network growth:

$$\begin{array}{c} \pi \left(v_{i,t},v_{j,t}\right)=\left[\frac{1}{2}\pi^{p}\left(v_{i,t},v_{j,t}\right)+\frac{1}{2}\pi^{h}\left(v_{i,t},v_{j,t}\right)+\epsilon_{ij,t}\right]*I_{ij,t}\;v_{i},v_{j}\in V,i\neq j \end{array}$$ (2)

where the network growth is driven by ranks of score *π*(*v*_*i*,*t*_, *v*_*j*,*t*_) of any node pair through mutual evaluation concerned about their preferences. It is calculated as the sum of preferential attachment score *π*^*p*^(*v*_*i*,*t*_, *v*_*j*,*t*_), the homophily score *π*^*h*^(*v*_*i*,*t*_, *v*_*j*,*t*_) and the random interference $\epsilon_{ij,t}∼N\left(0,\sigma^{2}\right)$ that follows a random normal distribution. *I*_*ij*,*t*_(*η*) is a binary vector that denotes the an encounters or zero encounters of this node pair with 1 or 0 dependent on the encounter rate *η* ∈ [0, 1].

The preferential attachment score *π*^*p*^(*v*_*i*,*t*_, *v*_*j*,*t*_) incorporates the preference **p**(*v*_*i*,*t*_) for the other nodes with higher/lower feature values for **f**(*v*_*i*,*t*_):

$$\begin{array}{c} \begin{array}{c} \pi^{p}\left(v_{i,t},v_{j,t}\right)=\frac{1}{2l}f{\left(v_{j,t}\right)}^{\tau }\left(p\left(v_{i,t}\right)⊙w^{p}\left(v_{i,t}\right)\right)+\frac{1}{2l}f{\left(v_{i,t}\right)}^{\tau }\left(p\left(v_{j,t}\right)⊙w^{p}\left(v_{j,t}\right)\right)+1 \end{array} \end{array}$$ (3)

The homophily score *π*^*h*^(*v*_*i*,*t*_, *v*_*j*,*t*_) incorporates the preference *p*(*v*_*i*,*t*_) for the other nodes with similar/dissimilar feature values *f*(*v*_*i*,*t*_):

$$\begin{array}{c} \begin{array}{ccc} \pi^{h}\left(v_{i,t},v_{j,t}\right) & = & \frac{1}{2l}{\left|f\left(v_{i,t}\right)-f\left(v_{j,t}\right)\right|}^{\tau }\left(h\left(v_{i,t}\right)⊙w^{h}\left(v_{i,t}\right)\right)\\ & + & \frac{1}{2l}{\left|f\left(v_{i,t}\right)-f\left(v_{j,t}\right)\right|}^{\tau }\left(h\left(v_{j,t}\right)⊙w^{h}\left(v_{j,t}\right)\right)+1 \end{array} \end{array}$$ (4)

The score list *Π*_*t*_ = {*π*(*v*_*i*,*t*_, *v*_(_*j*, *t*))|*v*_*i*,*t*_, *v*_*i*,*t*_ ∈ *V*, *i* ≠ *j*} stays frozen with fixed nodes and node attributes (features and sDNA). The DT-CNS simulator connects λ_*e*,*t*_ pairs of nodes based on the score ranks and generates a static network to represent discrete interactions at this frozen time point, with an edge intensity of

$$\begin{array}{c} \gamma \left(v_{i,t},v_{j,t}\right)=\left\{ \begin{array}{cc} \frac{\pi (v_{i,t},v_{j},t)+2l}{4l} & \text{if}\;\lambda_{e,t}\;\text{is}\;\text{reached};\\ 0 & \text{if}\;\text{else}. \end{array}\right. \end{array}$$ (5)

The score list *Π*_*t*_ changes with any node feature change/preference mutation. This may result in changes of node pair ranks and their connections, finally leading to network evolution.

#### Process and process dynamics

In this study, we also investigate the Susceptible-Infected (SI) epidemic spreading process on social networks based on the seed selection strategy and the transmission rules concerning the epidemic transmissibility impacted by different conditions (See Background).

**Fig 3:**
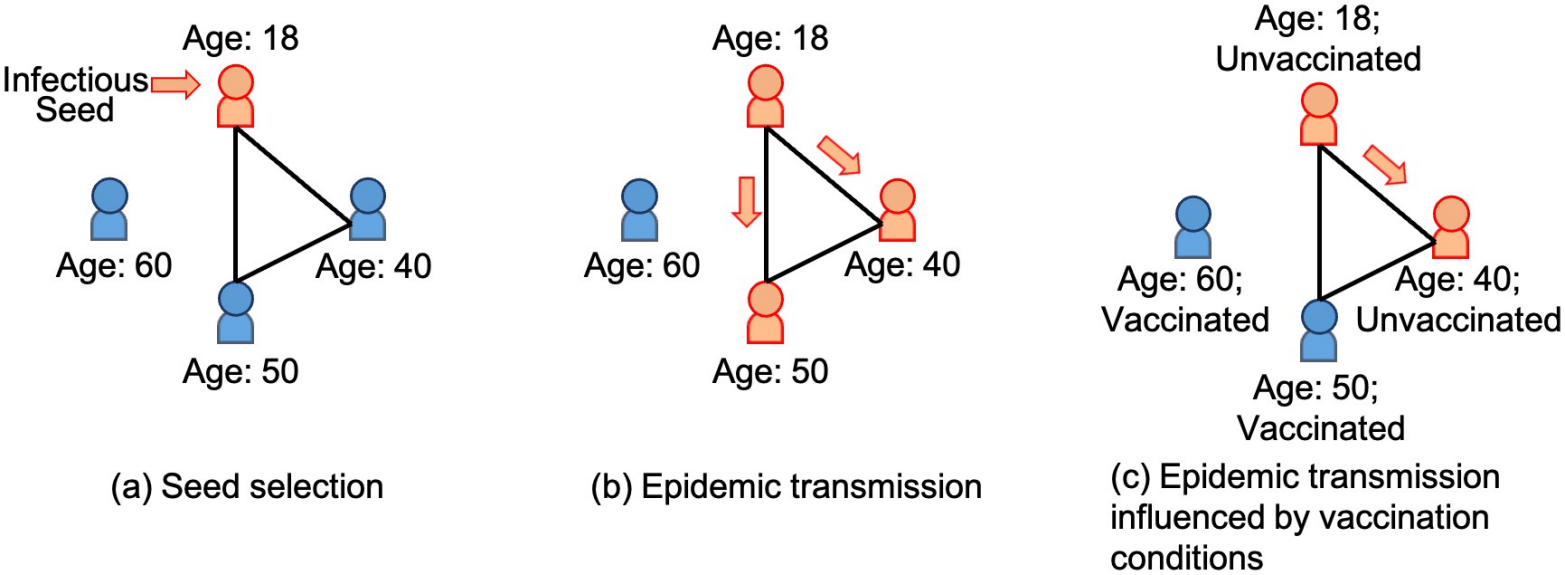
An illustrative example of epidemic spreading process, including seed selection (a) and epidemic transmission with and without the impact of nodes’ vaccination conditions (b) and (c). We assume the epidemic transmissibility to be 100% and the nodes, after vaccination, are immune to the epidemic spread.

We use the following example to illustrate the process dynamics. As shown in Fig 3(a), we select the first infectious node in the social network, the youngest node, aged 18. In Fig 3(b), we assume an epidemic transmissibility at 100%, implying an epidemic spread at 100% probability given exposure to the infectious node. The epidemic spread from the seed node (aged 18) to its neighbours (node aged 40 and node aged 50). In Fig 3(c), we consider the impact of vaccination conditions, where vaccinated nodes are immune from epidemic spread. As a result, the node aged 50 keeps healthy in epidemic spread due to vaccination. The epidemic spreading patterns get even more complex and reach the real-world epidemic spreading characteristics when we consider different seed selection strategies, changes in transmissibilities and influences from heterogeneous nodes’ conditions. Therefore, we propose the following process dynamics to approach and extend the simulations of real-world epidemic-spreading phenomena by incorporating various seed selection strategies and transmission rules.

Process Dynamics drives the spreading process P = {Ω, *Pr*_*t*_, **B**_*t*_, **R**_*t*_} on the networks and is defined by the following elements: seed selection strategy Ω, transmission probability *Pr*_*t*_ given the epidemic spread’s transmissibility *β*_*t*_, the nodes’ adoptability **B**_*t*_ = {**b**(*v*_*i*,*t*_)|*v*_*i*,*t*_ ∈ *V*_*t*_} and the resulting infection status **R**_*t*_ = {**r**(*v*_*i*,*t*_)|*v*_*i*,*t*_ ∈ *V*_*t*_}.

The seed selection strategy identifies the first contagious nodes at the beginning of epidemic spread. The seed selection strategy Ω = {*ω*_1_, *ω*_2_, ⋯} for single/multiple seeds in the initial/sequential stages is defined with a set of rules *ω*_*t*_:

$$\begin{array}{c} \omega_{t}\left(s_{t},w_{t}^{s},k_{t}\right) \end{array}$$ (6)

where *k*_*t*_ represents the number of seeds selected at time *t*. **s**_*t*_ is a *l*-length vector that determines the seed preference for node features **f**(*v*_*i*,*t*_) with values: 0, 1 or −1, each representing no preference, preference for higher/lower feature values, respectively. This is accompanied by a *l*-length weighting vector $w_{t}^{s}$, including weights of preference within range (0, 1]. The first *k*_*t*_ seeds are selected at time *t*, dependent on the descending ranks of node score *ϵ*(*v*_*i*,*t*_):

$$\begin{array}{c} \epsilon \left(v_{i,t}\right)=f{\left(v_{i,t}\right)}^{\tau }\left(s⊙w^{s}\right) \end{array}$$ (7)

For example, we can build a seed selection strategy based on node popularity preference. In this context, popular nodes, featured with significant node degrees, are preferred and assigned high score ranks, finally selected as the contagious seeds.

The seed selection results in the nodes’ infection status **r**(*v*_*i*,*t*_), a *m*-length vector that determines whether the node is infected or not with binary values of 0 or 1. Its transition from the previous infection status **r**(*v*_*i*,*t*−1_) to the current status **r**(*v*_*i*,*t*_) is dependent on the node adoptability *α*(*v*_*i*,*t*_), also termed as the Process DNA (pDNA):

$$\begin{array}{c} b\left(v_{i,t}\right)=\left\{c\left(v_{i,t}\right),\Theta \left(v_{i,t}\right),w^{c}\left(v_{i,t}\right)\right\} \end{array}$$ (8)

The pDNA is represented through three *q*-length vectors: **c**(*v*_*i*,*t*_) = [*c*_1_(*v*_*i*,*t*_), ⋯, *c*_*q*_(*v*_*i*,*t*_)] specifies the *q* independent conditions of node *v*_*i*,*t*_ considering the node infection status **r**(*v*_*i*,*t*_) and the node features **f**(*v*_*i*,*t*_). The independent conditions presented in **c**(*v*_*i*,*t*_) identify various individual characteristics that pose each individual at infection risk and determine how the epidemic spreads. As an example, there is an epidemic spread between males with physical contact. In this context, the independent conditions include gender (male/female) and exposure (with/without physical contact).

Θ(*v*_*i*,*t*_) = [*θ*_1_(*v*_*i*,*t*_), ⋯, *θ*_*q*_(*v*_*i*,*t*_)] represents the adoptability-thresholds to be considered when evaluating each node condition; and $w^{c}\left(v_{i,t}\right)=\left[w_{1}^{c}\left(v_{i,t}\right),\ldots ,w_{q}^{c}\left(v_{i,t}\right)\right]$ includes the multiplier effect of node adoptability on state transition, within the value range (0, 1].

With the pDNA **b**(*v*_*i*,*t*_) and the transmissibility *β*_*t*_ of the spread, the transition probability *Pr*(**r**(*v*_*i*,*t*_)|**r**(*v*_*i*,*t*+Δ*t*_)) can be calculated as:

$$\begin{array}{c} Pr\left(r\left(v_{i,t}|r\left(v_{i,t-\Delta t}\right)\right)\right)=\beta_{t}*\prod_{j=1}^{q}{\left[w_{j}^{c}\left(v_{i,t}\right)\right]}^{\delta_{c_{j}\left(v_{i,t}\right),\theta_{j}\left(v_{i,t}\right)}} \end{array}$$ (9)

where *δ* is a Kronecker function that determines, with binary values between 0 and 1, whether a specific condition *c*_*j*_(*v*_*i*,*t*_) for node *v*_*i*,*t*_ meets the threshold *θ*_*j*_(*v*_*i*,*t*_). To be more specific, $\delta_{c_{j}(v_{i,t}),\theta_{j}(v_{i,t})}=0$ indicates the elimination of the multiplier effect $w_{j}^{c}\left(v_{i,t}\right)$, while given $\delta_{c_{j}(v_{i,t}),\theta_{j}(v_{i,t})}=1$, $w_{j}^{c}\left(v_{i,t}\right)\to 0$ indicates full resilience of the node.

### An evaluation protocol on faithfulness

Below, we propose an evaluation protocol to formally capture different interpretations of faithfulness under the constraints of observability that vary with the no/complete/partial ground-truth scenarios.

#### Complexity

We first evaluate DT-CNSs conceptually by identifying the complexity generations of DT-CNSs based on three key characteristics, including the evolvability of DT-CNSs, interrelations within DT-CNSs and the interplay between the DT-CNSs and the reality (See Introduction; See Fig 1). From generation 1 to generation 5, the DT-CNS components, including networks and processes on networks, get increasingly complex by modelling instantaneous and interrelated changes. The temporal scale ranges from static (fixed over time), evolving (captured in snapshots) to temporal (continuous in real-time). Meanwhile, the DT-CNSs start to interplay with reality through real-time data acquisition and feedback between these two counterparts. This involves the linkage, one-way/two-way real-time information flow, between the physical real world and the virtual DT-CNS model, enabling the DT-CNSs to approach a DT.

Emergent patterns observed in DT-CNSs and their complexity are the starting point of quantitative evaluation. The evaluation metrics of the emergent patterns existing in DT-CNSs in the Network dimension and the Process dimension (See Table 1) respectively focus on the characteristics of network topology, network attributes, the transmissibility of the spread and the infection occurrence. As DT-CNSs evolve from generation 1 to generation 5, these quantitative metrics start to change over time with a narrowed time gap between each observation and serve as important indicators to evaluate the similarity between DT-CNSs and reality.

**Table 1:** Measures of complex patterns emerging from DT-CNS representation.

CNS components		Pinpoint	Local	Global
Network	Topology	Node [47], Edge [48]	Triad [45], Quadrangle [56], Community [63–65], Clustering coefficient [46], Closure coefficient [46]	Degree distribution [43], Shortest path length [43], Modularity [43, 44], Assortativity, Betweenness, Closeness.
	Attribute	Node attribute [66, 67], Edge attribute [67, 68]		Feature distribution, Hill numbers [69]
Process	Spread	Transmissibility to nodes	Transmissibility to groups	Transmissibility to the population
	Infection	Infected nodes	Infected groups	Infected population

These measures range from (i) the pinpoint level connected to the individual component of the network/process (e.g. a node/edge added/removed over time; transmissibility to a node,together with its resulting infection status); (ii) the local level concerned with the local interactions among grouped individuals (e.g. clustering coefficient employed to assess local structure); and (iii) the global level concerned with the emergent global characteristics resulting from the interactions within the population (e.g. degree distribution based on the number of edges connected to each node; Hill numbers that describe the diversity of the population (nodes) based on node attributes).

#### Performance

Performance of modelling DT-CNS dynamics, given the quantified complexity of DT-CNS, can be evaluated in respect to different requirements in terms of the DT-CNS patterns’ reproducibility, the corresponding similarity between the DT-CNS patterns with a target state (e.g. the target network topology required in link prediction tasks; etc.) as well as efficiency (See Table 2).

**Table 2:** Measures used to assess performance of simulation, prediction and/or control models of DT-CNS dynamics.

Requirements		Measures
Similarity	Pinpoint	Precision [48, 57], Recall [70], Area Under the Precision–Recall (AUPR) curve [71], Receiver Operating Characteristic (ROC) curves, Area Under the ROC (AUC) [72], Geometric Mean of AUC and PRAUC (GMAUC) [73], Error Rate [74], SumD [75], Kendall’s Tau Coefficient (KTC) [76], Micro/Macro/Weighted Average Precision/Recall/F1 Score [77, 78]
	Local	Kullback-Leibler divergence [59], Jensen–Shannon divergence [59],Manhattan distance [79], Canberra Distance [79], Euclidean distance [58], Matusita distance [58]
	Global	Kullback-Leibler divergence [59], Jensen–Shannon divergence [59], Manhattan distance [79], Canberra Distance [79], Euclidean distance [58], Matusita distance [58], Earth Mover’s Distance [80], Similarity metrics respectively based on entropy distance, spectral distance, modality distance, cosine of the angle between two graphs [81].
Efficiency	Data processing	Time delays compared with the real time data flow [60]
	Simulation/Modelling	Runtime of the simulation/modelling [19, 61]
Reproducibility	Same data	Yes/No
	Same statistics	Kullback-Leibler divergence [59],Jensen–Shannon divergence [59]
	Same phenomena	*ω*-index [82], City organization index [83]

Based on the measures describing complex emerging patterns in a DT-CNS, similarity between the created and the target DT-CNS patterns also ranges from the pinpoint level, the local level, to the global level.

The pinpoint level similarity is generally considered in prediction tasks, like the link prediction and its evaluation with precision [72] where the similarity is interpreted in the context of the links predicted vs links actually appearing. In contrast, the local and the global similarity are generally employed in the simulation optimisation of DT-CNSs toward the target network topological features.

Efficiency required for the DT-CNS dynamics involves the discussion from two perspectives: the efficiency of data processing, concerned with the time delays compared with the real-time data flow [60], as well as the efficiency of modelling, connected to the runtime of DT-CNSs [19, 61].

Reproducibility requires the equivalent results of the same tasks [25]. It varies with the increasingly demanding levels of equivalence in the recreated data, ranging from the same phenomena and statistic distribution to the same data. Current DT-CNSs generally fall into the category of the same statistics. For example, the scale-free network model generates networks that share the same node degree distributions based on a given parameter set.

#### Faithfulness

Faithfulness of DT-CNS representation and modelling is defined and evaluated based on the model performance under the respective observability constraints. This is because we can only evaluate the faithfulness of a DT-CNS regarding the observable real-world information. Therefore,under different observability constraints, simulation-based, data-driven, and hybrid DT-CNSs each poses different requirements of efficiency, reproducibility and similarity, which results in different meanings of faithfulness (See Table 3).

**Table 3:** Requirement of a faithful DT-CNS representation and modelling under observability constraints. The solid and the hollow circle each represents the fulfilment or partial fulfilment of the corresponding requirement for each type of modelling.

Requirements		Simulation-based DT-CNSs	Data-driven DT-CNSs	Hybrid DT-CNSs
Similarity	Pinpoint		▶	▷
	Local	▶		▷
	Global	▶		▷
Efficiency	Data processing		▶	▷
	Simulation/Modelling	▶	▶	▶
Reproducibility	Same data		▶	
	Same statistics	▶		▶
	Same phenomena	▶		▶

As is shown in Table 3, the solid and the hollow circle each represents the fulfilment or partial fulfilment of the corresponding requirement for each type of modelling. The data-driven DT-CNSs, as well as the data-driven components of hybrid DT-CNSs, generally require pinpoint similarity with the real data and efficiency in data processing and modelling. The simulation-based DT-CNSs and the simulation components of Hybrid DT-CNSs need global and local similarity, as well as efficiency of a simulation process. In terms of reproducibility, data-driven DT-CNSs, in the ideal case, are required to generate the same data, mimicking the reality. In contrast, for the simulation-based DT-CNSs and the hybrid DT-CNSs, the same statistics and phenomena are needed and sufficient. For example, some data-driven DT-CNSs employ network embedding methods in node classification tasks [19, 84, 85]. They require a pinpoint level of similarity with ground-truth information about network topology and attributes, data processing and modelling efficiency, and reproducible classification results for validation and comparative analysis. The simulation-based DT-CNSs, such as the scale-free network models, simulate realistic networks based on the assumptions of interaction rules—the preferential attachment to popular nodes [86, 87]. They require a global level similarity and the reproducibility of the statistics that measure the required network characteristics, such as the degree distributions reproduced by the same scale-free network model. Some hybrid DT-CNSs employ real social networks and simulate the spreading processes on these networks [4]. They first represents the real networks with pinpoint level similarity and then, based on these networks, simulate spreading processes with reproducible spreading results.

### Optimisation towards a Digital Twin

**Fig 4:**
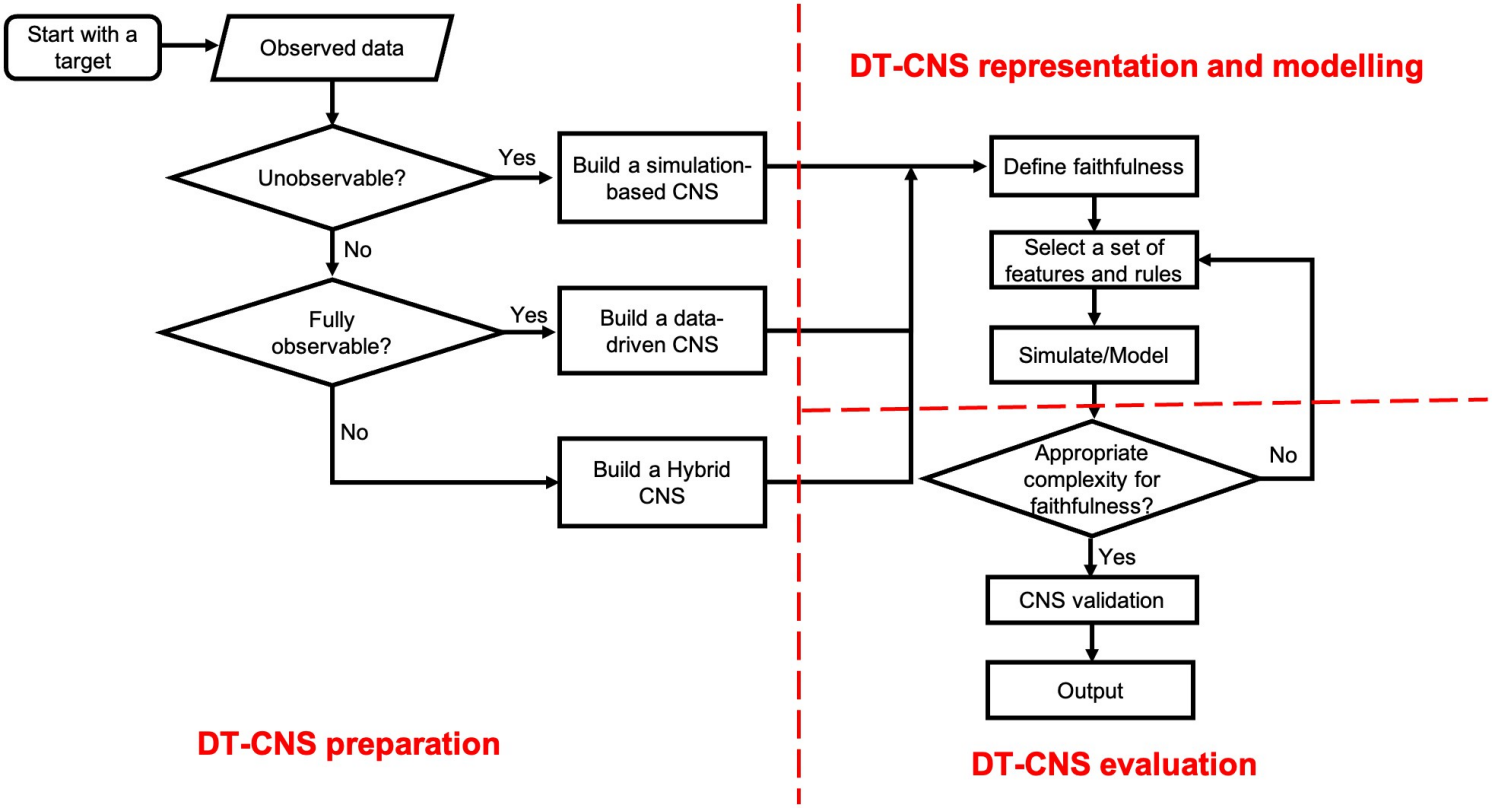
The procedures of building Digital Twin-Oriented Complex Networked Systems.

Under observability constraints, we optimise the DT-CNSs for an appropriate level of complexity, which enables a minimised information loss with a limited number of variables. We also create a set of modelling procedures to drive the optimisation of DT-CNSs considering different DT-CNS types and their required faithfulness given the level of observability (See Fig 4).

As shown in Fig 4, the devise and optimisation of the DT-CNS towards a DT go through the DT-CNS preparation, DT-CNS representation and modelling, and the DT-CNS evaluation procedures. We prepare a DT-CNS by targeting a specific state of real systems and, under the observability constraints, deciding the DT-CNS type (See An extendable DT-CNS framework). After that, we determine the faithfulness needed for a DT-CNS under the evaluation protocol proposed in An Evaluation Protocol on Faithfulness. In addition, we devise and optimise an extendable DT-CNS towards a DT through an appropriate selection of features (See Table 4) and rules (See Table 5).

**Table 4:** Examples related to features and the conditions in the DT-CNS representation and modelling for the social networked systems in an epidemic outbreak.

Features&Conditions				Data-driven	Simulation-based
Network	Node	Ascribed features	Continuous	Height, weight, etc.	Normal distribution, uniform distribution, etc.
			Discrete	Age, etc.	Poisson distribution, etc.
			Categorical	Gender, nationality, group, etc	Bernoulli distribution
		Topological features	Continuous	Node clustering coefficient, centrality, etc.	
			Discrete	Node degree, shortest path lengths, etc.	
	Edge	Ascribed features	Continuous	Intensity of contact/relationship, etc.	Normal distribution, uniform distribution, etc.
			Discrete		Poisson distribution, etc.
			Categorical	Relations, Types of contact, etc.	Bernoulli distribution
		Topological features	Continuous	Duration of contact/relationships, etc.	
			Discrete	Frequency of contact/Rating of relationships, etc.	
Process	Seed	Resulting conditions	Categorical	Infection status.	
	Spread	Ascribed conditions	Categorical	Immunity, susceptibility, etc.	
		Resulting conditions	Categorical	Infection status, an exposure, multiple exposure, etc.	

**Table 5:** Examples of possible rules governing the interactions, seed selection and epidemic transmission in DT-CNS representation and modelling.

DT-CNS component	Rule		Parameters	Value range	Description	Terminology
Network	Interaction rule	Preferential attachment	**p**(*v*_*i*,*t*_)	{−1, 0, 1}	Preference for larger/smaller feature values	social DNA
			**w**^*p*^(*v*_*i*,*t*_)	(0, 1]	Corresponding weight pf preference	
		Homophily	**h**(*v*_*i*,*t*_)	{−1, 0, 1}	Preference for similar/dissimilar features	
			**w**^*h*^(*v*_*i*,*t*_)	(0, 1]	Corresponding weight of preference	
Process	Transmission rule	Transmissibility	*β*(*v*_*i*,*t*_)	[0, 1]	Transmissibility of epidemic spread	transmissibility
		Node adoptability	Θ(*v*_*i*,*t*_)	{0, 1}	Adoptability threshold for each condition.	process DNA
			**w**^*c*^(*v*_*i*,*t*_)	(0, 1]	The multiplier effect on transmissibility when meeting the threshold	
	Seed selection	Seed preference	**s**_*t*_	{−1, 0, 1}	seed preference for node features	seed strategy
			$w_{t}^{s}$	(0, 1]	Corresponding weight of preference	
		Seed number	*k*_*t*_	(0, 1]	Number of seeds	

#### Features

Features in the DT-CNS context represent the attributes and the topological characteristics of nodes and edges in DT-CNSs. As is shown in Table 4, the network dimension has the ascribed and topological features, each resulting in and from the network growth. The process dimension focuses on the ascribed and the resulting conditions that change transmissibility and influence the spreading process. They are ascribed or resulting conditions dependent on whether they exist before or after the spread. For example, in social networks where epidemic spreads, nodes with specific ascribed features can have different susceptibilities given an epidemic exposure, such as gender and age. The epidemic spread can also result in conditions such as the states of infected, immune and susceptible. These resulting conditions also change the susceptibility of nodes in the next round of epidemic spread.

#### Rules

Rules are used to govern the behaviours of nodes in the DT-CNSs and they can be prescribed or learned from data. Those rules drive the network growth and the propagation of the spreading process based on various DT-CNS features. Rules that are considered in the framework are presented in Table 5. We parameterise the network growth with the social DNA vector based on preferential attachment and homophily rules. In addition, we model the spreading process through seed selection and the process DNA vector for node adoptability. The experiments on DT-CNSs in this study investigate the network growth based on the interaction rules presented in Table 5. The seed selection strategy and the transmission rules in Table 5 are part of the design but will be developed in the future research.

#### Trade-offs

Trade-offs between different requirements occur over the entire modelling and evaluation process to achieve highest possible faithfulness. These requirements involve the evaluation of DT-CNSs with different measures, respectively, considering the three aspects: similarity, efficiency and reproducibility. However, constrained by the available DT-CNS measures and modelling techniques for multi-objective optimisation, we make trade-offs in the modelling and evaluation process (See An Evaluation Protocol on Faithfulness) including:

- single-objective optimisation that combines different measures of one of these aspects: similarity, efficiency or reproducibility
- multi-objective optimisation considering all three aspects: similarity, efficiency and reproducibility
- minimised information loss for faithfulness and minimum number of variables under the constraint of observability

Current studies combine different similarity measures to calculate the loss function for an optimised DT-CNS [34]. Exceptionally, [29] proposes a comparative directed graph to compare the similarity of DT-CNSs based on the sum of different ranks considering different similarity measures.

The integration and trade-offs between similarity, efficiency and reproducibility emerge as a research gap under the proposed evaluation criteria. The data-driven scenarios have focused on minimised information loss and a minimum number of variables for years, which involves the discussion on feature selection/extraction and model validation. However, the features in the context of DT-CNS, simulation-based, data-driven or hybrid, differ between those scenarios due to the obseravability challenges and the simulation of informative and realistic features for DT-CNSs call for in-depth study in the future.

Finally, to validate the performance of a DT-CNS under the evaluation criteria, data-driven DT-CNSs, with more concerns related to the minimised information loss and the reproducible representation accuracy, can employ the cross-validation approaches through data split and repeated experiments. In contrast, for the simulation-based and hybrid DT-CNSs with partial observability, DT-CNS ensembles that create a series of DT-CNS representations with quantifiable, reproducible and realistic characteristicsare expected to prove the validity of DT-CNSs.

#### Extensions

We provide one of the possible pathways for extending the proposed DT-CNSs with increasing complex dynamics towards the DT of a real-world social networked system. This development path progresses referring to the DT-CNSs generations (See Fig 1, Tables 6 and 7) and gradually enables the evolvability in dynamics, interconnections between evolutionary dynamics and the interplay between the DT-CNSs and the reality.

**Table 6:** Complexity generations of DT-CNS from generation 1 to generation 5 (the ultimate goal of a DT) with increasing complexity levels, concerning the evovability of networks and processes on networks, their interrelations as well as their interplay with reality through the real-time information flow, including the one-way flow from reality to DT-CNS and the two-way flow between the reality and DT-CNSs.

Generation	DT-CNSs			Linkage		Reality
	Network	Process	Interrelation	Real-time Information Flow		Decision-Maker
				One-way	Two-way	
1	Static	Static	×	×	×	Non-reactive
2	Static	Evolving	×	×	×	Non-reactive
	Evolving	Static	×	×	×	Non-reactive
3	Evolving	Evolving	✓	×	×	Non-reactive
4	Temporal	Temporal	✓	✓	×	Non-reactive
5	Temporal	Temporal	✓	✓	✓	Reactive

**Table 7:** Development path of DT-CNS dynamics from generation 1 to generation 5 (the ultimate goal of a DT) with increasing complexity levels, concerning the interaction rules, transmission rules, and the real-world decision-making dynamics that are linked to the generation 5 DT-CNSs in a real-time feedback loop.

Generation	DT-CNSs		Reality
	Interaction Rule	Transmission Rule	Decision-Making
1	Non-Evolutionary	Non-Evolutionary	×
2	Non-Evolutionary	Evolutionary	×
	Evolutionary	Non-Evolutionary	×
3	Co-Evolutionary		×
4	Co-Evolutionary and Real-time Updatable		×
5	Co-Evolutionary and Real-time Updatable		

Generation 1 DT-CNSs, as shown in Table 6 and Fig 1, focus on static social networks and epidemic spreading processes with a static (fixed) transmissibility. In Table 7 and as proposed in this study, the generation 1 DT-CNSs of social networked systems model the static social networks based on heterogeneous node features (**f**(*v*_*i*,*t*_)) and non-evolutionary interaction rules (nodes’ preferences for features **p**(*v*_*i*,*t*_) and the corresponding preference weights **w**^*p*^(*v*_*i*,*t*_), as well as nodes’ preferences for feature differences **h**(*v*_*i*,*t*_) and the corresponding preference weights **w**^*h*^(*v*_*i*,*t*_); See Table 5)). In addition, we model the epidemic spreading phenomena as a non-evolutionary SI process based on an assumption/observation of seed selections (Ω), heterogeneous epidemic conditions (**c**(*v*_*i*,*t*_)) and a fixed transmissibility (*β*_*t*_).

Generation 2 DT-CNSs focus on the evolving social network changes and the evolving epidemic spreading process changes without considering their interrelations (See Table 6 and Fig 1). Generation 1 DT-CNSs, proposed in this study, can be extended to the generation 2 DT-CNSs by introducing evolutionary interaction rules and transmission rules to the modelling framework (See Table 7). This involves nodes’ preference mutation and spread’s transmissibility mutation over time.

Generation 3 DT-CNSs focus on the evolving epidemic spreading processes on the evolving social networks and consider the interrelations between those evolving dynamics (See Table 6 and Fig 1). Generation 2 DT-CNSs, based on evolutionary interaction rules and transmission rules (preference mutation and transmissibility mutation), can progress to generation 3 when allowing the interaction rules (preferences) and the transmission rules (transmissibility) to co-evolve under the impact of the social networks and the epidemic spreading process (See Table 7). For example, the nodes mutate their preferences to avoid the infection risks that result from the epidemic spread. Meanwhile, the epidemic’s transmissibility can mutate faster when it propagates through more nodes due to nodes’ social interactions.

Generation 4 DT-CNSs focus on the temporal epidemic spreading processes on temporal social networks. They model the interrelated temporal changes in DT-CNSs based on the real-time data acquisition from reality and the simultaneous model updates (See Table 6 and Fig 1). Generation 3 DT-CNSs, based on the co-evolutionary interaction rules and transmission rules (co-evolving preferences and transmissibility), can be extended to generation 4 when these co-evolutionary dynamics can be continuously updated according to the real-time information flow from reality to DT-CNSs (See Table 7). Generation 4 DT-CNSs approach the generation 5 DT-CNSs (DTs) when the simulation results of DT-CNSs feedback to reality in a real-time way and influence the real-world decision-making processes of those reactive decision makers (See Tables 6 and 7). In generation 5 DT-CNSs (DTs), the DT-CNS dynamics (interaction rules and transmission rules) and the real-world decision-making dynamics (e.g. policy-making) co-evolve and update simultaneously Fig 1). For example, the policy-maker creates a generation 5 DT-CNS of the social networked systems in an epidemic outbreak to investigate the possible impact of different epidemic mitigation policies, such as vaccination, isolation, etc. The generation 5 DT-CNS simulations provide feedback to the policy-maker and serve as a reference for policy-making.

## Results

In this section, we conduct experiments on simulation-based DT-CNSs to investigate the disaster resilience of different social networks considering the infection occurrences on these networks in an epidemic outbreak. We conduct a comparative analysis of simulation-based DT-CNSs driven by selected rules (preferential attachment and homophily) and distributions of the ascribed feature—age. We take the degree distribution of a scale-free network (composed of 90 nodes and 1400 edges, built with Barabasi-Albert network model [28]) as the target for the simulation of the static networks. To simulate the social networks, we assume 90 nodes, 1400 edges and an encounter rate of 0.8 for the respective node pairs. A simulation-based dynamic process takes place on the networks without changing its parameters over the temporal scale. We evaluate these models in terms of complexity and their faithfulness, considering the global level similarity (measured by the difference between degree distributions), simulation efficiency, and reproducibility of the same statistics.

We respectively term the involved simulation-based DT-CNSs as *DT* − *CNS*_*U*_, *DT* − *CNS*_*B*_, *DT* − *CNS*_*I*_, *DT* − *CNS*_*L*_, *DT* − *CNS*_*R*_ and *DT* − *CNS*^*H*+^, *DT* − *CNS*^*H*−^*DT* − *CNS*^*P*+^, *DT* − *CNS*^*P*−^ and *DT* − *CNS*^*HP*^, to characterise the DT-CNS paradigms with uniform (*U*), bell (*B*), inverse bell (*I*), left-skewed (*L*) and right-skewed (*R*) age distributions, respectively driven by preferential attachment to larger feature values (*P*+), preferential attachment to smaller feature values (*P*−), heterophily (*H*+), homophily (*H*−), and rules that optimally incorporate the above principles for a target degree distribution.

### Simulation-based networks

In this section, we build the simulation-based networks with 90 nodes, 1400 edges and a single feature—age, ascribed to each node. We presume that the age involved in disaster resilience range between [0, 90] while categorising the age groups every ten years [88].

#### Feature

**Fig 5:**
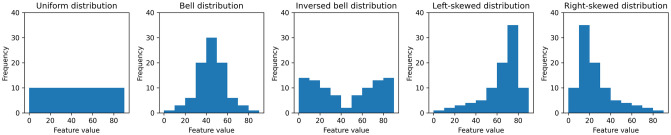
The age distributions used in the experiments.

We simulate the uniform, the bell, the inverse bell, the left-skewed and the right-skewed shape distributions for a fixed number of nodes allocated in each age group. We also introduce uncertainty through a randomly generated age value within the range required by each age group (See Fig 5).

In Fig 5, the numbers of nodes in each age group vary depending on distribution. There are 10 nodes evenly allocated for each age group in a uniform distribution. In contrast, more/fewer nodes are in their 40s in a bell/inverse bell distribution than in other age groups. In a left-skewed distribution, most nodes are over 60, and vice versa for a right-skewed distribution. We evaluate the age diversity with the Hill number, which measures the effective number of equally abundant species (age groups) [51, 89]. The varying diversity levels differentiate the respective age distributions, indicating the population’s age differences and resulting in different connection patterns.

**Fig 6:**
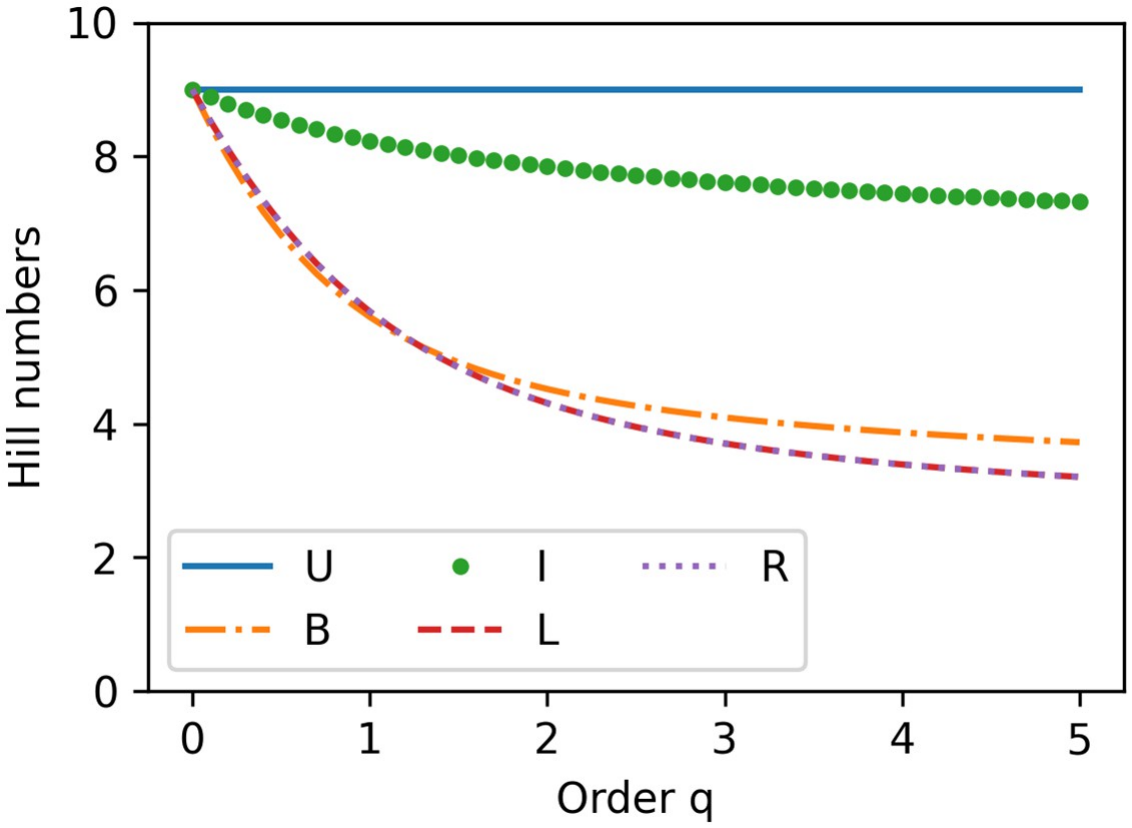
The Hill number for each age distribution.

In Fig 6, we calculate the Hill numbers with an order value that determines the sensitivity to the relative frequencies of the species (age groups), valuing between 0 and 5. A higher order value indicates a higher sensitivity level to the relative number of nodes allocated in each age group. With an order value of 0, the Hill numbers get insensitive to the abundance of the respective species (age groups) and keep at 9 the number of age groups for all the age distributions [4]. With the same order value, the higher Hill number indicates higher diversity. As the age distributions transform from the uniform, inverse bell, bell to skewed age distributions, their Hill numbers decrease, indicating less age diversity.

#### Preference

Given the age feature and the different rules of network formation, including the preferential attachment to larger feature values (P+), the preferential attachment to smaller feature values (P-), the heterophily (H+), the homophily (H-) as well as the optimised combination of both the preferential attachment and the homophily principles (PH), the scores of node pairs vary a lot and indicate different strengths of relationships. The parameter set-ups of preferences and weights of preferences is included in Table A in the S1 Appendix.

#### Network topology

**Fig 7:**
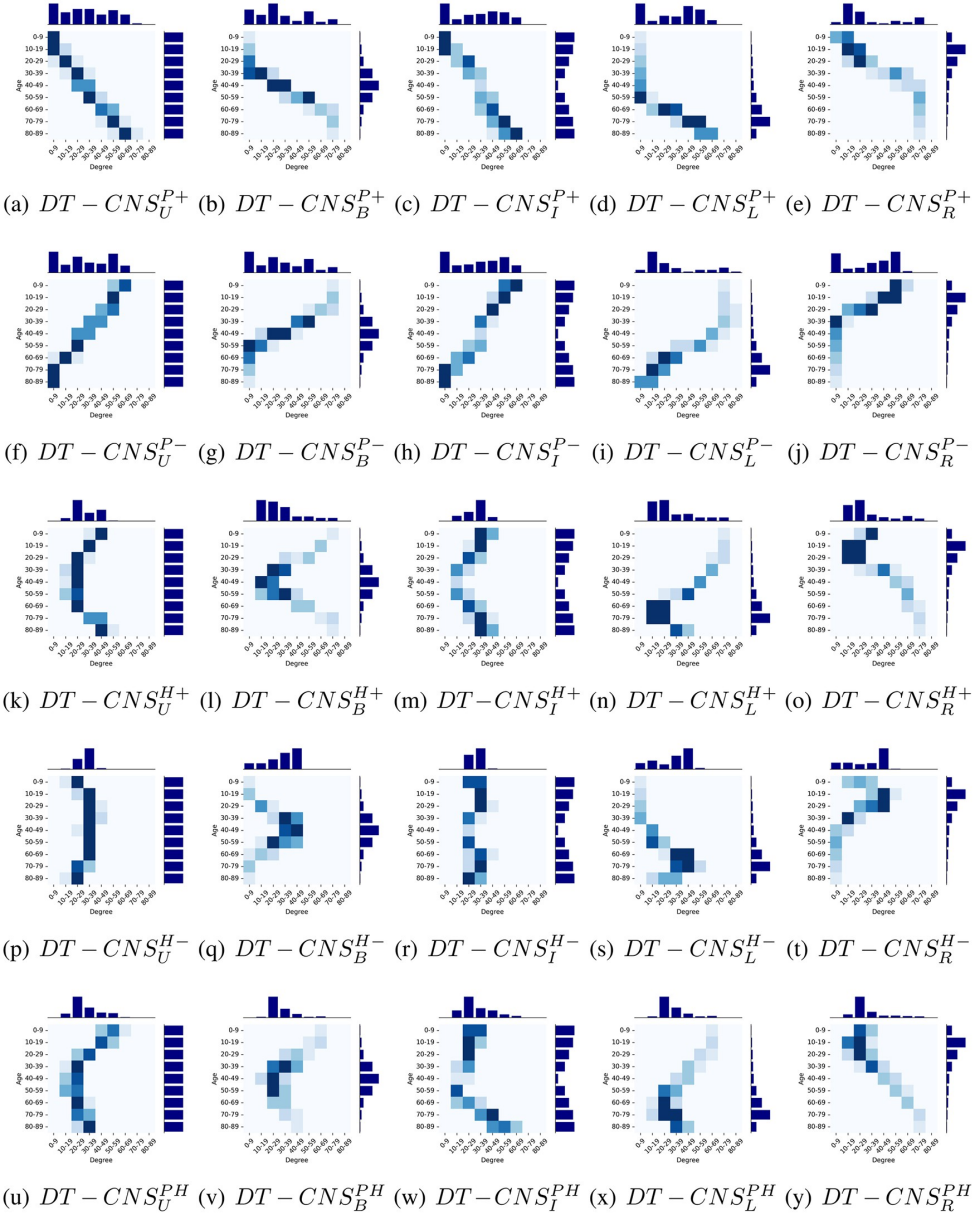
The age and degree distributions of social networks generated by DT-CNSs based different features and rules.

**Fig 8:**
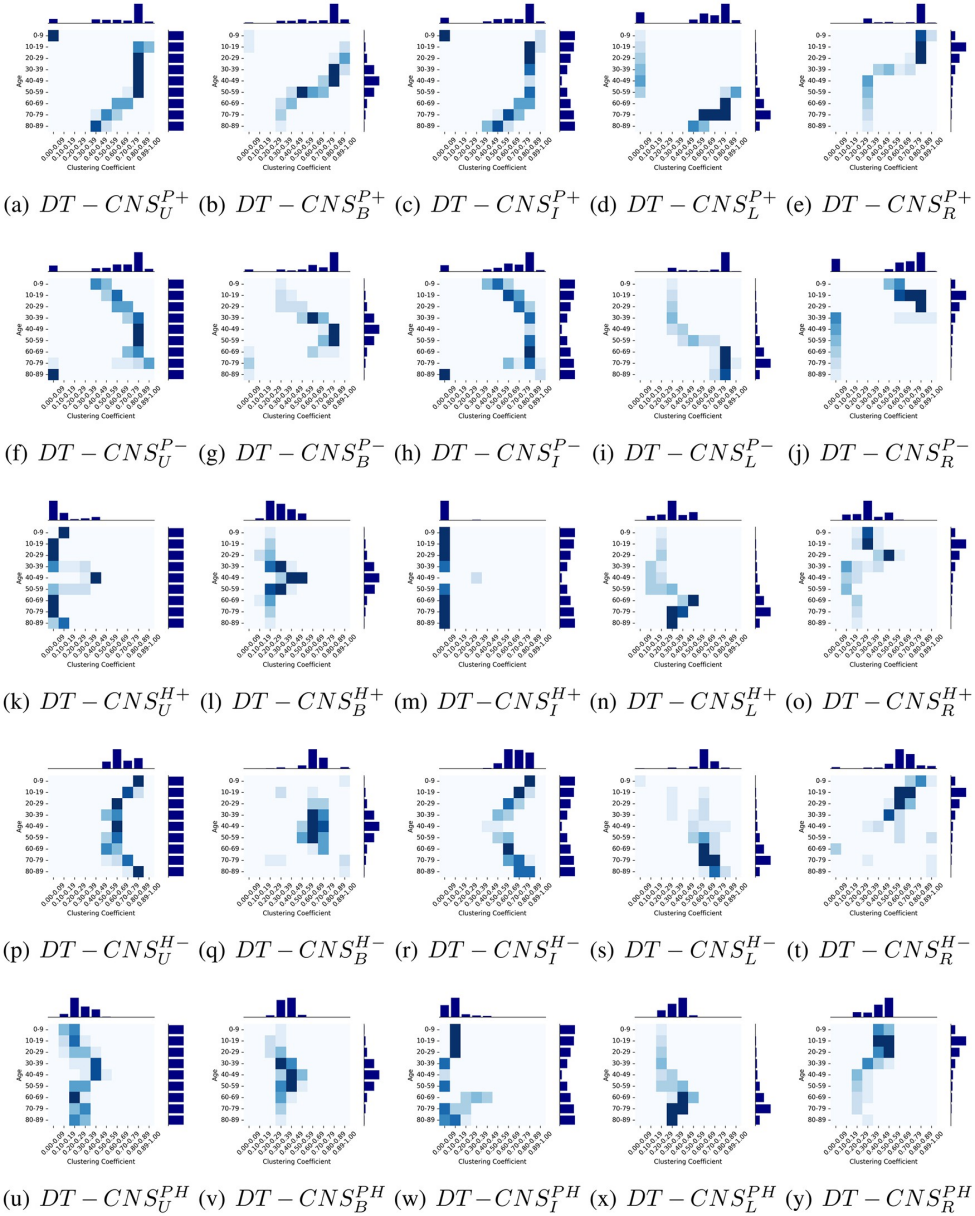
The age and clustering coefficient distributions of social networks generated by DT-CNSs based different features and rules.

Given the same threshold factor λ_*e*_ = 1400, that determines the number of edges, the simulated networks are correspondingly characterised with varying network topology (See Figs 7 and 8).

**Degree Distribution** is a distribution of node degrees in a given network [43]. As shown in Table 8, the node degree of the target network fluctuates around the average value of 31.11 with a standard deviation of 12.90, ranging from 6.00 to 70.00.

**Table 8:** Topological information of the networks generated with the DT-CNSs.

Features	Rules	Nodes		Node Degree				Clustering coefficient				Shortest path length				
		Connected	Unconnected	Avg.	Std.	Max.	Min.	Avg.	Std.	Max.	Min.	Fake Paths	Avg.	Std.	Max.	Min.
Target Network		90	0	31.11	12.90	70	6	0.47	0.08	0.79	0.33	0	1.65	0.48	3	1
Uniform	*P*+	80	10	31.11	21.20	70	0	0.66	0.27	1.00	0.00	845	20.22	36.09	90	1
	*P*−	81	9	31.11	21.48	68	0	0.67	0.28	1.00	0.00	765	18.47	34.76	90	1
	*H*+	90	0	31.11	9.64	52	16	0.12	0.14	0.45	0.00	0	1.69	0.54	3	1
	*H*−	90	0	31.11	6.16	43	19	0.69	0.09	0.89	0.57	0	2.05	0.98	5	1
	*PH*	90	0	31.11*	11.56*	63*	13*	0.31	0.09	0.52	0.16	0*	1.65*	0.48*	3*	1*
Bell	*P*+	89	1	31.11	21.34	77	0	0.73	0.21	1.00	0.00	89	3.61	13.03	90	1
	*P*−	87	3	31.11	21.50	75	0	0.70	0.22	0.89	0.00	264	7.46	21.93	90	1
	*H*+	90	0	31.11	15.70	73	13	0.36	0.11	0.57	0.16	0.00	1.65	0.48	2	1
	*H*−	90	0	31.11	12.85	47	2	0.68	0.10	1.00	0.30	0	2.12	1.16	8	1
	*PH*	90	0	31.11	10.32	68	17	0.40	0.06	0.52	0.28	0	1.65	0.48	2	1
Inverse bell	*P*+	79	11	31.11	21.24	66	0	0.66	0.28	1.00	0.00	924	21.96	37.26	90	1
	*P*−	80	10	31.11	21.54	67	0	0.66	0.29	1.00	0.00	845	20.23	36.08	90	1
	*H*+	90	0	31.11	8.03	42	12	0.01	0.05	0.36	0.00	0	1.79	0.67	3	1
	*H*−	90	0	31.11	4.71	40	22	0.72	0.10	0.88	0.48	0	2.18	1.07	5	1
	*PH*	90	0	31.11	12.63	63	10	0.14	0.10	0.47	0.00	0	1.66	0.5	3	1
Left skewed	*P*+	75	15	31.11	21.34	64	0	0.61	0.32	1.00	0.00	1230	28.69	40.82	90	1
	*P*−	90	0	31.11	22.47	81	7	0.74	0.19	0.90	0.30	0	1.65	0.48	2	1
	*H*+	90	0	31.11	16.80	73	14	0.37	0.11	0.54	0.15	0	1.65	0.48	2	1
	*H*−	90	0	31.11	14.50	50	1	0.66	0.11	0.83	0.00	0	2.42	1.67	9	1
	*PH*	90	0	31.11	11.25	67	15	0.39*	0.08*	0.53*	0.23*	0	1.65	0.48	2	1
Right skewed	*P*+	90	0	31.11	22.41	79	7	0.73	0.2	0.93	0.31	0	1.65	0.48	2	1
	*P*−	73	17	31.11	21.69	62	0	0.59	0.34	1.00	0.00	1377	31.92	42.04	90	1
	*H*+	90	0	31.11	17.07	74	15	0.36	0.12	0.6	0.14	0	1.65	0.48	2	1
	*H*−	90	0	31.11	15.48	51	1	0.67	0.17	1.00	0.00	504	13.06	29.21	90	1
	*PH*	90	0	31.11	14.24	74	14	0.46	0.09	0.58	0.25	0	1.65	0.48	2	1

Fig 7 shows age and degree distributions with a heatmap and two histogram plots for age (See Fig 5) and node degree (See Table 8). We find that the node degrees of each age group varies with the interaction rules related to preferences for age. For example, the *DT* − *CNS*^*P*+^ models, built on positive preferential attachment to age, tend to have more older nodes connected with each other and those older nodes have higher node degrees than in case of other models. Similarly, the *DT* − *CNS*^*H*−^ models built on homophily when it comes to age wire more nodes within the same age groups, enabling the nodes in age groups with more members to have higher node degrees.

Given the same interaction rules, the node degrees of respective age groups also vary with the shapes of age distributions. The symmetric and asymmetric shape distributions can lead to a different trend of node degree changes with age change. For example, for $DT\;-\;CNS_{B}^{H+}$ based on the *H*+ (heterophily) rule and the bell-shape age distribution, the node degree first decreases from [70 − 79] to [10 − 19] when the node age transits from [0−9] to [40 − 49], which then increases from [10 − 19] to [70 − 79] as the node age transits from [50 − 59] to [60 − 89]. This contrasts with the case of $DT\;-\;CNS_{L}^{H+}$, where the node degree first decreases from [70 − 79] to [10 − 19] when the node age transits from [0 − 9] to [60 − 69], which then increases from [10 − 19] to [40 − 49] as the node age transits from [70 − 79] to [80 − 89]. This phenomenon can be caused by the number of nodes in each age group, as nodes in sparse age groups are preferred and connected with those dissimilar others in dense age groups. This inevitably leads to higher node degrees for these sparse age groups. In contrast, the dense age groups have lower node degrees as they prefer the sparse age groups and have limit connections with these preferred nodes. In addition, when we optimise the combined preferences considering both preferential attachment and homophily (*DT* − *CNS*^*PH*^ models), the shapes of degree distributions approach the shape of a power-law distribution. This indicates that the introduction of features and the optimisation of corresponding preferences enable better achievement of the target states of networks.

**Clustering Coefficient** describes the probability of a node’s neighbours to be connected. Its value is between 0 and 1 [43]. As shown in Table 8, in the target network, clustering coefficient fluctuates around an average value of 0.47 with a standard deviation of 0.08, ranging from 0.79 to 0.33.

Fig 8 shows age and clustering coefficient distributions with a heatmap and two histogram plots for age (See Fig 5) and clustering coefficient (See Table 8). We find that nodes in age groups with bigger number of people with connection preferences for a similar group of nodes tend to cluster with a higher clustering coefficient. For example, the young and middle-aged nodes in the *DT* − *CNS*^*P*+^ models, built on positive preferential attachment to age, prefer to be connected with old nodes, where these nodes cluster around the popular old nodes. Similarly, the nodes in *DT* − *CNS*^*H*−^ models, where interactions are based on homophily phenomenon, like to be connected with similar others. This generally leads to a higher clustering coefficient than in the case of other DT-CNSs. However, nodes in *DT* − *CNS*^*H*+^ models, driven by heterophily phenomenon, prefer to be connected with dissimilar others. They generally have lower clustering coefficients than in the case of other DT-CNSs because they select different nodes to connect and do not cluster around the same node. When we optimise the combined preferences considering both preferential attachment and homophily, the shapes of clustering coefficient distributions vary with age distributions. As nodes tend to connect with similar and old nodes (referring to the optimised preferences for both preferential attachment and homophily in Table A in the S1 Appendix), the old and dense age groups tend to have higher clustering coefficient. Among them, the $DT\;-\;CNS_{U}^{PH}$ and the $DT\;-\;CNS_{I}^{PH}$ models characterised with more even age group allocations generate a clustering coefficient with a power-law shape because less nodes fulfil one of the requirements for larger age values and denser age groups, which induces a lower clustering coefficient in the respective cases.

**Shortest Path Length** between two nodes describes the number of edges along the shortest path between a pair of nodes [43]. As shown in Table 8, the shortest path length in the target network fluctuates around an average value of 1, 65 with a standard deviation of 0.48, ranging from 1.00 to 3.00.

**Fig 9:**
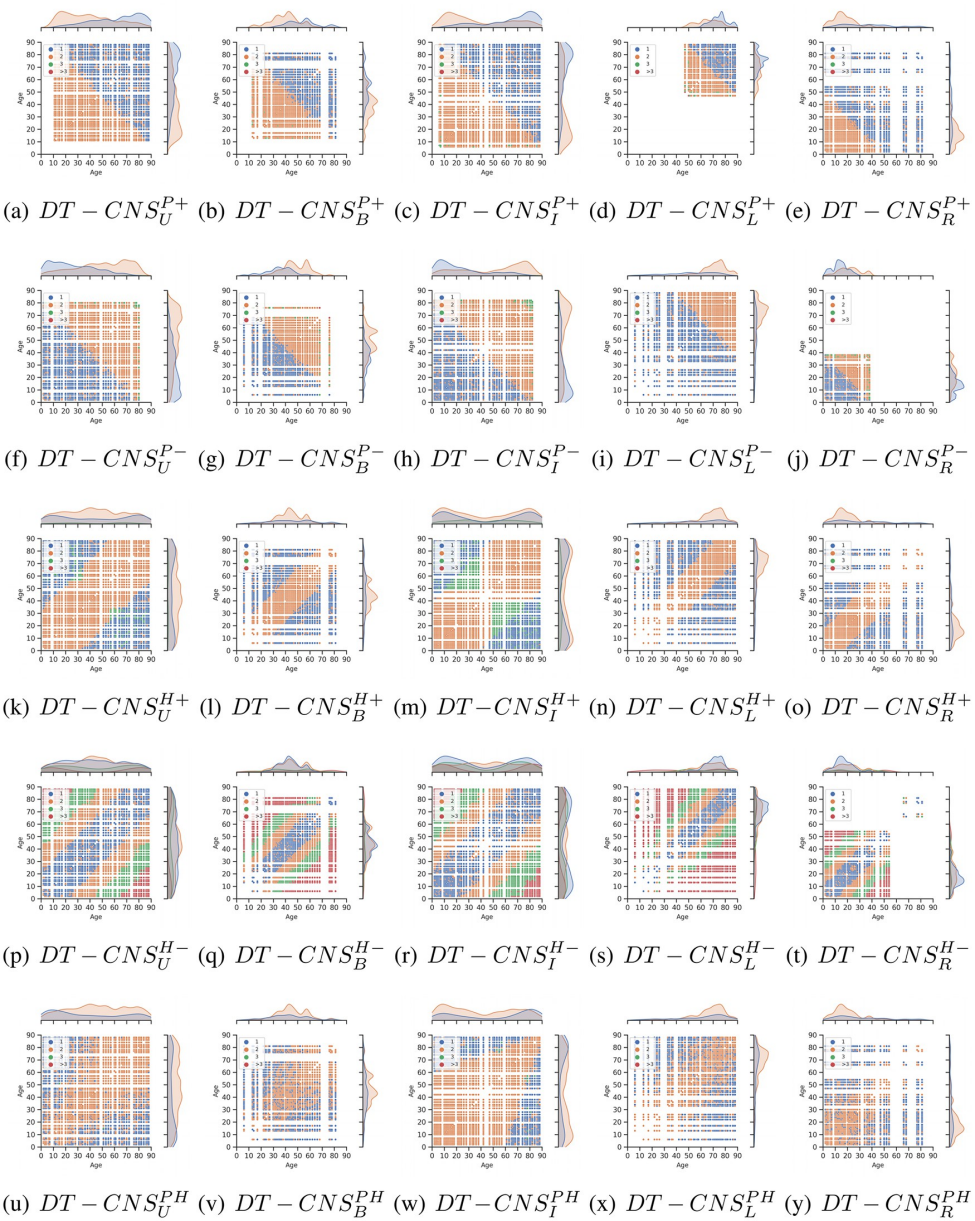
The age and shortest path length distributions between connected nodes of social networks based on different features and rules.

Fig 9 shows age and shortest path length distributions with a scatter plot and two same Kernel Density Estimation (KDE) plots for shortest path length referring to the age values (See Fig 5 for age distributions and Table 8 for information related to the shortest path length). Specifically, in Fig 9, we present the shortest path length at 1, 2, 3 and over 3 between the connected nodes with blue, orange, green and red dots. The x-axis and y-axis represent age values and the scattered dots represent the shortest path lengths between given pair of nodes of given ages. We find that the shortest path lengths between the nodes are generated by all the *DT* − *CNS* models range between 1 and 3 except for the *DT* − *CNS*^*H*−^ models built on the *H*− (homophily) rule. This is because in *DT* − *CNS*^*H*−^ models, similar nodes cluster within the similar age groups, leading to longer paths to connect the dissimilar nodes. For example, in the $DT-CNS_{L}^{H-}$ model, which is built with a left-skewed age distribution and the homophily principle, a large number of paths between 0 − 9 and 80 − 89 age groups have value over 3 as a high proportion of young nodes in the left-skewed age distribution cluster together without many direct connections with the nodes for which the age range is 89 − 90. In addition, the distribution of the shortest path lengths is also highly related to the preference principles. The age groups with preferred features have shorter paths to other nodes. For example, the nodes which are old in *DT* − *CNS*^*P*+^ models have more direct connections with others and the shortest paths of length of 1 due to the fact that they have preferred value of feacture age. When we optimise the combined preferences considering both preferential attachment and homophily, due to the complex interaction rules, the shapes of shortest path lengths distributions vary greatly with age distributions and become very hard to interpret. This indicates that the increasing dynamics level depreciates the network patterns’ interpretability but enables the creation of more complex network and more faithful scenarios.

**Summary Statistics,** related to the target network and the simulated networks, are presented in Table 8. It incorporates information related to the degree distributions, clustering coefficient distributions and shortest path length distributions. In addition, we use JS divergence to evaluate the distances between these network patterns with that of the target network (See Tab. A in the S2 Appendix). More specifically, the most similar network patterns are identified with marker *. Compared with the target network, the network simulated by the $DT\;-\;CNS_{U}^{PH}$ model generates the most similar degree distribution and shortest path length distribution. $DT\;-\;CNS_{L}^{PH}$ model generates the most similar clustering coefficient distribution. This indicates that network models, driven by different features and the optimised interaction rules, approach different characteristics of the target network. These differences challenge the evaluation and development of DT-CNS models, remaining a research gap to be addressed in future studies. However, in our experiments, we only focus on the degree distribution of target network and investigate how heterogeneous features and rules influence the performance of DTCNSs in recreating similar degree distributions.

Table 8 shows the number of connected and unconnected nodes and the information about the degree distributions, clustering coefficient distributions and the shortest path length distributions that vary depending on the values of the features and rules employed. Generally, all the nodes get connected with the *DT* − *CNS*^*H*+^, *DT* − *CNS*^*H*−^ and *DT* − *CNS*^*PH*^ paradigms. *DT* − *CNS*^*P*+^ or *DT* − *CNS*^*P*−^ paradigms, driven by preferential attachment to old/young ages, result in some unconnected old/young nodes given any age distribution due to the strong preference for young/old ages. In contrast, *DT* − *CNS*^*H*+^, *DT* − *CNS*^*H*−^ and *DT* − *CNS*^*PH*^ paradigms consider the effect of similar or dissimilar ages, where all nodes can find similar/dissimilar others within/across age groups and connect with them. The node degree distributions for each DT-CNS paradigm share the same mean values but have different standard deviation, maximum and minimum values. Degree distributions, generated by *DT* − *CNS*^*H*−^ and *DT* − *CNS*^*PH*^ paradigms, generally have smaller standard deviations and maximum values. In contrast, *DT* − *CNS*^*P*+^ and *DT* − *CNS*^*P*−^ paradigms have larger ones. The maximum degree of the respective paradigms are similar with the number of connected nodes, which indicates that the most popular node tends to directly connect with most of the connected nodes (resulting in power law node degree distribution). The node clustering coefficient distributions for *DT* − *CNS*^*P*+^ and *DT* − *CNS*^*P*−^ models based on preferential attachment principles and *DT* − *CNS*^*H*−^ models built on homophily generally have larger average values, standard deviations and maximum values than other models, which results from the dense clusters created by preferred nodes (See Fig 8). The shortest path length distributions are especially influenced by the number of unconnected nodes and the corresponding non-existing (’fake’) paths, which are assumed as the maximum path length plus one (i.e. 90). For the fully connected networks, the average shortest path length fluctuates around 2, given different age distributions and preferences. It increases significantly when there are fake paths for unconnected node pairs.

### Simulation-based processes

In this section, we build simulation-based processes to analyse disaster resilience given different network structures under the influence of various age distributions. The susceptible nodes get infected given exposure with fixed transmissibility rate, while infected nodes get infectious over time without recovery. The epidemic spread on the networks propagates one step (edge) away for each time step.

#### Seed selection

We investigate the most severe case of epidemic explosion by assuming a single seed selection in the initial stage based on the largest node degree (See Table A in S3 Appendix) and allow the epidemic propagation for 5 time steps within 6 steps (edges) away from the seed (first infection). Within this time and distance range, most of the connected nodes finally get infected as they are directly/indirectly connected with the seed. As shown in Table 8 and Table A in S3 Appendix, almost all the connected nodes are connected with the seed node, resulting in a higher infection risk and the full infection. The seeds selected for all the modelling paradigms generally fall in an age group which are preferred by much denser age groups. For example, the old (young) nodes are preferred by all other nodes in *DT* − *CNS*^*P*+^ paradigms (*DT* − *CNS*^*P*−^ paradigms) and thus have the largest number of connections, which almost directly connect all the connected nodes in the respective model (See Table 8)

#### Infection occurrence

The infection occurrence refers to the proportion of infected nodes to the entire population within a specific distance from the seed. Given the infectious seed, we simulate an epidemic spreading process based on different transmissibilities (ranging in [0.2, 0.4, 0.6, 0.8]) and investigate their impact on the infection occurrence.

We represent the number of infection occurrences within specific number of steps (edges away) from the first infection in Fig. A in S4 Appendix. As we simulate the epidemic spread for 6 time steps and allow the epidemic propagation by one step (edge) for each time step, nodes within 6 steps away from the first infection are likely to be infected and thus have infection risks in the epidemic simulations. Nodes out of this range will not be exposed to the infection risks and thus some of the nodes stay uninfected despite the increase of transmissibility. The infection occurrence generally stays the same given any transmissibility between 0.4 and 1.0. The detailed analysis of Fig. A can be seen in S4 Appendix.

**Fig 10:**
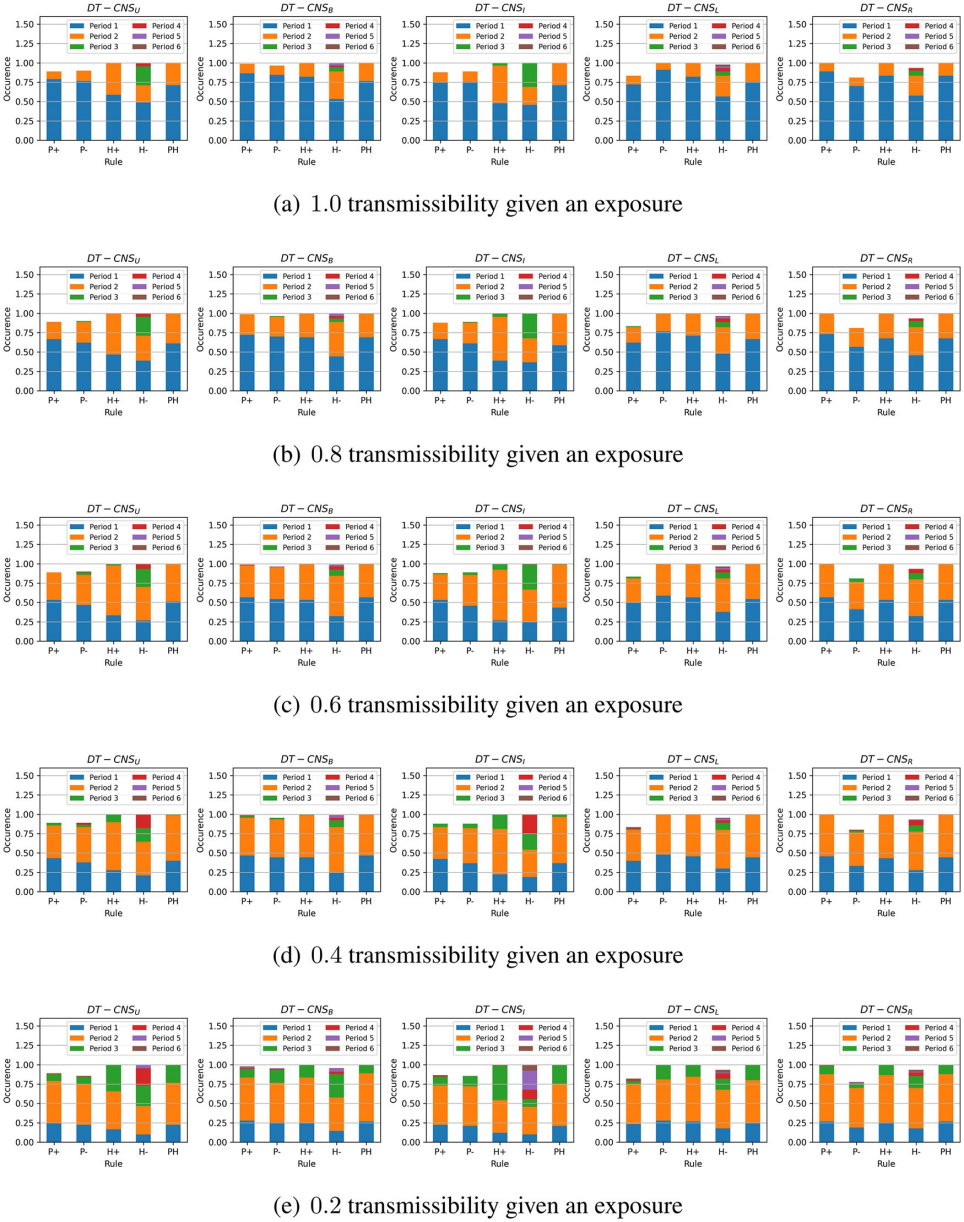
The infection occurrence over time with different transmissibilities given an exposure.

With decreasing transmissibility of the epidemic spread given the exposure, it takes more time steps to achieve the infection status shown in Fig. A in S4 Appendix. In Fig 10, the number of infected nodes within the first time step (represented as Period 1) gets smaller when the transmissibility decreases from 1.0 to 0.2, while the infections increase within Period 2 and Period 3. Given a smaller transmissibility, the nodes are less likely to get infected in Period 1 and thus stay healthy. However, these healthy nodes in Period 2 and Period 3 get exposed to more infected neighbours and finally get infected, leading to a delayed increase in infection numbers. The decrease of transmissibility results in increase of time needed to infect all the nodes and in the same time gives more time to react to the disaster. Compared with the other models, *DT* − *CNS*^*H*−^ models, driven by the homophily principle, have smaller infection occurrences despite different transmissibilities. In addition, the infection occurrences for *DT* − *CNS*^*H*−^ models also increase slowly compared to other models. This is because the seed node clusters around a limited number of similar nodes (See Table A in S3 Appendix). This leads to a social distancing between the infectious seed and dissimilar nodes. The increase in transmissibility does not bridge the social distance between the seed node and the dissimilar nodes. Therefore, for *DT* − *CNS*^*H*−^ models, it still takes more time for the epidemic spread to reach and infect these nodes. For infection occurrences in various age distributions over different time steps, *DT* − *CNS*_*I*_ generally has the fewest infections within the first time step. This also results from limited connections between the seed node and others (See Table A in S3 Appendix). The abovementioned phenomenon indicates that the increase in transmissibility can have a greater impact on networks that densely clusters around the seed node.

#### People at risk

We introduce the People at Risk (*PaR*) measure to have an intuitive representation of the proportion of people at risk of a disaster:

$$\begin{array}{c} PaR\left(T,D\right)=\frac{\sum_{v_{i,t}\in V_{t}}r\left(v_{i,t}\right)\delta_{l(v_{i,t},s_{t}),D}\delta_{t,T}}{N},\;D\leq T \end{array}$$ (10)

which is defined as *PaR*(*T*, *D*) to help identify the easiest-to-be-infected proportion of the population in the limited time [0, *T*] and the space within the distance [0, *D*] away from the seed. *δ*_*t*,*T*_ and $\delta_{\text{l}(v_{i,t},s_{t}),D}$ each represents the Kronecker functions related to time *t* and the distance $\text{l}(v_{i,t},s_{t})$ between node *v*_*i*,*t*_ and seed **s**_*t*_. *N* represents the number of nodes.

**Fig 11:**
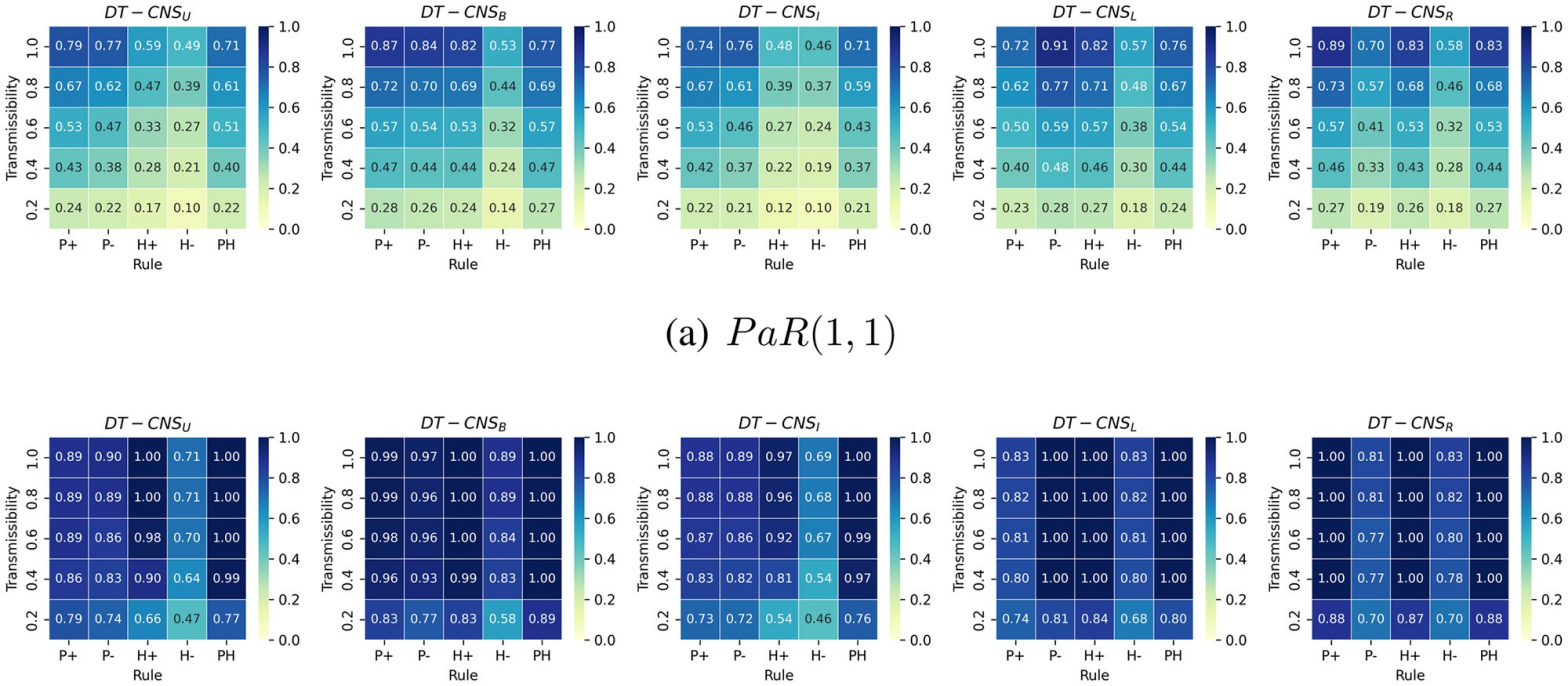
The *PaR*(1, 1) and *PaR*(2, 2) values with different transmissibilities, age distributions and rules of network formation.

Fig 11 shows the *PaR*(1, 1) and the *PaR*(2, 2) values that vary with transmissibilities, rules of network formation and the age feature distributions. First, the *PaR*(1, 1) decreases as transmissibility decreases. *DT* − *CNS*^*P*+^, *DT* − *CNS*^*P*−^, *DT* − *CNS*^*H*+^ and *DT* − *CNS*^*PH*^ generally have a higher *PaR*(1, 1), and this is due to the higher number of nodes connected to the seed (See Fig. A in S4 Appendix., where there are more occurrences of infections within the distance 1). In addition, *DT* − *CNS*^*U*^ and *DT* − *CNS*^*I*^, due to a higher age diversity, have smaller *PaR*(1, 1) values. The *PaR*(2, 2) values increase significantly as the transmission propagates one step further. Almost all the connected nodes get infected within two time steps, despite the differences in transmissibilities (See Fig 10). This can be caused by the significant number of direct connections between the popular seed node and others (See Table 8). Similar with the case of *PaR*(1, 1) values, the *PaR*(2, 2) values for the *DT* − *CNS*^*H*−^ models are also smaller than for the other models, resulting from the limited number of connections with the seed node (See Table 8). The above-mentioned differences between *PaR*(1, 1) and *PaR*(22) values indicate the necessity of epidemic control in primary time and distance to the seed node.

To better understand the diversity of age features and their influence on the infection status, we respectively calculate the *PaR*(1, 1) of each age group based on different transmissibilities, age groups and rules (See Figs A–E in S5 Appendix). We also identify the age group where the epidemic starts with a green arrow in the corresponding *PaR*(1, 1) figures. The figures and the corresponding analysis are in S5 Appendix, which mainly presents three phenomena. First, the *PaR*(1, 1) increases with the increasing transmissibility. This indicates an increasing level of difficulty to resist an epidemic outbreak given increased transmissibility, because a higher *PaR*(1, 1) value represents a higher proportion of infected nodes and more resources that are needed to keep the epidemic under control (See Fig. A in S5 Appendix). Second, the density in age groups influences the corresponding *PaR*(1, 1) values. Denser age groups, despite the differences and complexities in preferences, generally have higher infection risks and correspondingly lower resistance levels to the epidemic outbreak (See Fig. E in S5 Appendix). Third, the preference for old (young) age groups with positive (negative) preferential attachment to age, as represented by *P*+ (*P*−) rule, may pose the old (young) at higher risk (See Figs A and B in S5 Appendix). The *P*− (negative preferential attachment to age) and *H*+ (heterophily) rules lead to similar *PaR*(1, 1) values because, in both these cases, the young and dense age groups prefer to be connected with the seed node. Given the *H*− rule (preferences for similar ages) of network formation, fewer age groups get involved in the epidemic spreading process than the other rules since the homophily effect limits the interactions with the seed node to similar age groups (See Fig. D in S5 Appendix).

### Faithfulness

**Fig 12:**
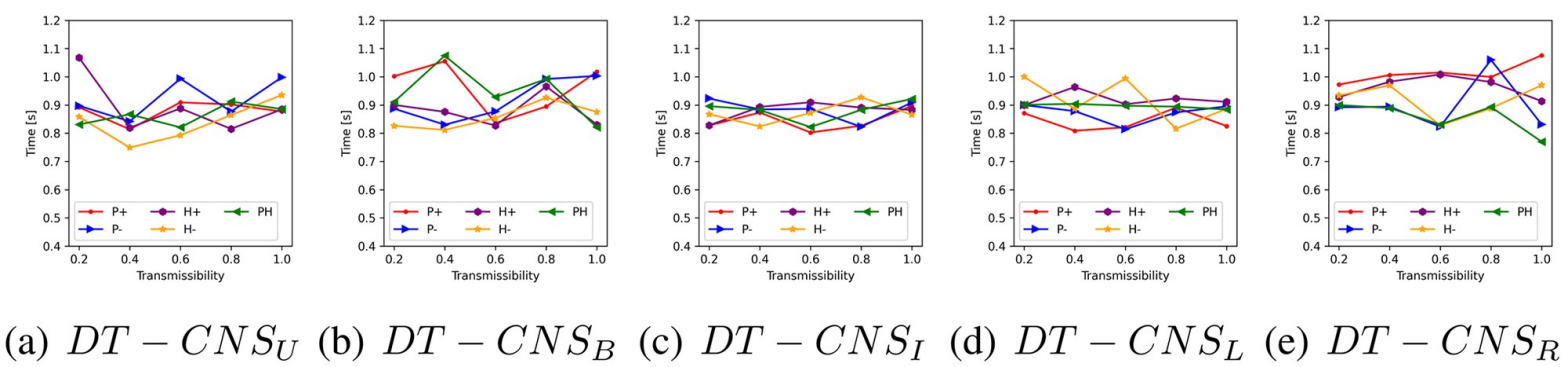
The runtime of simulation-based DT-CNS given different features and rules.

In this section, we evaluate the simulation-based DT-CNSs based on their network representations’ faithfulness. This involves the similarity of simulated network with the target network (see Table 9), the simulation runtime (see Fig 12) and the reproducible network statistics.

**Table 9:** The similarity of degree distributions with the target degree distribution, as measured by the Jensen–Shannon divergence.

Paradigms	*P*+	*P*−	*H*+	*H*−	*PH*
*DT* − *CNS*_*U*_	0.51	0.53	0.36*	0.43	0.26*#
*DT* − *CNS*_*B*_	0.50*	0.50*	0.45	0.50	0.27
*DT* − *CNS*_*I*_	0.56	0.54	0.46	0.42*	0.33#
*DT* − *CNS*_*L*_	0.56	0.53	0.49	0.53	0.28#
*DT* − *CNS*_*R*_	0.51	0.59	0.47	0.55	0.28#

We evaluate the similarity of simulated networks with the target network from a global perspective using the degree distribution. In addition, we apply the Jensen–Shannon divergence (JS divergence) [59] to measure the similarity of degree distributions. A smaller JS divergence indicates a higher similarity level. In Table 9, *DT* − *CNS*_*U*_, *DT* − *CNS*_*B*_ and *DT* − *CNS*_*I*_ paradigms, with more diverse age distributions where nodes are more evenly distributed in the age groups with a similar density, tend to generate more similar degree distributions than the paradigms built on other distributions (See the marker * in Table 9). *DT* − *CNS*^*PH*^ paradigms, with an optimised preference principles have smaller JS values (See the market # in Table 9). $DT\;-\;CNS_{U}^{PH}$ paradigm achieves the most similar degree distribution with the target. Therefore, the scale-free network pattern, described by its degree distribution from a global perspective, can be approached based on node features and the nodes’ preferences for connecting with others. The consideration of node features is a good way to built up the complexity of a model and approach the reality.

We employ the runtime of simulations to evaluate the efficiency of the *DT* − *CNS* paradigms, where, as is shown in Fig 12, the time spent on simulation fluctuates with varying transmissibility. The *DT* − *CNS*^*H*−^ paradigms generally have the lowest runtime due to the relatively lower node degrees and the resulting inactivity of epidemic spread. Overall and as expected, the diversity of age distributions and the different rules of network formation, as well as the resulting patterns from the epidemic spreading process, do not make much difference, indicating a similar efficiency level for all the involved *DT* − *CNS* paradigms.

Regarding reproducibility, we ensure the repetition of the same resulting patterns with the involved DT-CNSs by setting up a random seed to generate the same random distributions in the process of feature simulation, network formation and process simulation. These random distributions include (i) the uniform distribution that generates age values within an age group (e.g. U(20,29) for the [20–29] age group), (ii) the Bernoulli distribution B(0.08) that generates binary values to represent encounters of node pairs based on the 0.08 encounter rate in network simulation, (iii) the normal distribution $N(0,0.005^{2})$ that generates the random interference in the mutual evaluation between node pairs in the network simulation and (iv) the Bernoulli distribution that generates binary values to represent infections of nodes based on the aggregated infection risks from the exposures in process simulation.

### Summary of results

In this section, we summarise the experiment results on the simulation-based DT-CNSs in disaster resilience scenarios connected with the emerging network patterns and the infection status.

In the network dimension, the diversity of the population increases as the age distribution gets more even. Nodes at an older/younger age are more popular than others given *P*+ or *P*− rules. In contrast, nodes closer/further away from the average age are more popular with *H*+ or *H*− rules. The $DT\;-\;CNS_{U}^{PH}$ paradigm, based on the most diverse age distribution and driven by an optimised *PH* rule that prefers a younger and dissimilar age, better approaches the target scale-free network patterns. This indicates that introducing features and combining corresponding preferences enable the DT-CNSs to approach the target states better with a higher complexity level concerning the network attributes and dynamics. Our proposed DT-CNS modelling framework enables the extension of DT-CNSs with a flexible complexity levels and the evaluation of their respective distances to the target.

In the process dimension, we study the infection status in the disaster resilience scenario under the influence of network structures driven by various age features and interaction rules. DT-CNS driven by *P*+, *P*− and the *H*+ rules can generally achieve the most infections within two steps (edges) away from the seed. The decrease of transmissibility means that more time is needed to reach an infection peak, preserving time to react to the epidemic outbreak. DT-CNS paradigms driven by *P*+, *P*− or *H*+ rules tend to take less time for the final infection status. This is because the seed node has more direct connections with other nodes, in contrast with the nodes who connect with limited number of similar others under the influence of homophily effect. The heterogeneous features and respective preferences lead to complex network topologies, inducing different infection patterns that occur sooner or later due to various transmissibilities. Therefore, social networks have different infection risks/occurrences and, correspondingly, different resistance levels to an epidemic outbreak within a specific time, dependent on transmissibilities and network topologies driven by features and preferences. As a result, nodes with different features and preferences also suffer different infection risks, present different resistance levels to the epidemic and thus can be treated with different mitigation policies.

To promote disaster resilience, the diversity of the population and the interaction rules are important drivers of case-dependent policy-making. We employ the people at risk *PaR*(1, 1) to represent the proportion of the infected people to be treated by the policymakers with the highest priority. The experiment results suggest that larger transmissibility, a less diverse population, and the *P*+ (positive preferential attachment in age), *P*− (negative preferential attachment in age) and *H*+ (heterophily effect) rules contribute to a higher *PaR*(1, 1). Among each age group, the *P*+ and *P*− rules each lead to higher *PaR*(1, 1) for the older and the younger, while *H*+ and *H*− lead to clustering infection among several age groups due to their connections to similar and dissimilar others respectively. Therefore, the control of epidemic outbreak requires mitigation policies targeted at heterogeneous population. The age groups with higher *PaR* values given an upcoming epidemic outbreak should be vaccinated in priority to contain the spread of disease. The social networks characterised with higher *PaR* values within specific time and space require more stringent and urgent isolation policies when the epidemic spreads.

## Conclusion

This study proposes a modelling framework for DT-Oriented CNSs based on heterogeneous node features and interaction rules. We also create an evaluation protocol for a faithful representation of reality. Under the modelling framework and evaluation protocol, we conduct a case study on disaster resilience, where we build and compare the simulation-based DT-CNSs given various features and rules.

We build the DT-CNS modelling framework from a top-down perspective concerned with the network and the process dynamics (see An extendable DT-CNS framework). We first employ an inner-rule-based network model to represent the network faithfully and mimic its growth. This network model is built on heterogeneous features (ascribed and topological features—see Table 4) and various feature preference principles (homophily and preferential attachment principles—see Optimisation towards a Digital Twin). We also devise the (epidemic) process model based on a seed selection strategy and node adoptability, which all vary with specific conditions concerned with node features and the infection status. Based on these features and rules, the network growth and dynamic process are parameterised with sDNA and pDNA vectors, each incorporating the feature preferences and the consideration of conditions related to transmissibility.

We also propose an evaluation protocol on faithfulness concerned with similarity, efficiency and reproducibility (see An Evaluation Protocol on Faithfulness). Under this protocol, the definition of faithfulness varies with the simulation-based DT-CNSs, hybrid DT-CNSs and data-driven DT-CNSs due to the ground-truth availability. There are pinpoint-, local- and global-level similarity employed, each focusing on the network components, networks local structures, and the global network statistics. We also use the runtime of the simulation and/or modelling to measure the efficiency and evaluate the reproducibility of the same data, statistics or phenomena.

We finally conduct a case study on disaster resilience and conclude with three findings. First, the diversity of the population, people preferences with who they interact and a change of transmissibility can significantly influence infection patterns and serve as important indicators for policymakers in a disaster resilience scenario. Second, the complexity of network dynamics increases when more feature preference representation principles (e.g. preferential attachment and homophily) are introduced. Such an increase in complexity can improve the faithfulness of network representation by preserving necessary heterogeneous patterns observed in reality. Third, age diversity influences the network structures and induces the epidemic outbreak among specific people. This implies the necessity of targeting the easily infected populations with heterogeneous mitigation policies.

Our proposed modelling framework and evaluation protocol enable the extension of DT-CNSs with a flexible complexity level and the evaluation of their respective distances to the target. This modelling framework can also be employed in our future study to generate more realistic social networks by incorporating more complex and real-world information. Our simulation-based experiments on age features and related interaction rules indicate the complexity of real-world interactions and reveal the challenge of approaching reality where people’s features and preferences can be uncertain and, thus, even more complex. This poses a research question to be addressed in our future study: how to improve the expressive power of DT-CNSs for achieving more real-world like network representation?

## Supporting information

S1 Appendix The parameters of the DT-CNSs. (PDF)

S2 Appendix The JS divergence between the network patterns of simulated networks and the target network. (PDF)

S3 Appendix The single seed selection based on the largest node degree. (PDF)

S4 Appendix The infection occurrences within different distances to the seed node. (PDF)

S5 Appendix The *PaR*(1,1) of each age group based on different transmissibilities, age groups and rules. (PDF)

S1 Fig The infection occurrence within certain distance to the seed given different transmissibilities. (TIF)

S2 Fig The *PaR*(1,1) for each age group given different transmissibilities, age distributions and the *P*+ rule. (TIF)

S3 Fig The *PaR*(1,1) for each age group given different transmissibilities, age distributions and the *P*− rule. (TIF)

S4 Fig The *PaR*(1,1) for each age group given different transmissibilities, age distributions and the *H*+ rule. (TIF)

S5 Fig The *PaR*(1,1) for each age group given different transmissibilities, age distributions and the *H*− rule. (TIF)

S6 Fig The *PaR*(1,1) for each age group given different transmissibilities, age distributions and the *PH* rule. (TIF)
