# Supplementary material for: Towards Digital Twin-Oriented Complex Networked Systems: Introducing heterogeneous node features and interaction rules
Source: PLoS One. 2024 Jan 2;19(1):e0296426. doi: 10.1371/journal.pone.0296426 (PMC10760715; doi:10.1371/journal.pone.0296426)
Supplement: S1 Appendix — (PDF) [file pone.0296426.s001.pdf]

S1 Appendix.

We present the parameter set-ups for preferences and weights of preferences of the DT-CNSs in this study. In our experiments, we assume an encounter rate at 0.80 and a random interference that follows a random normal distribution $\mathbf{N}(0, 0.005^2)$. We optimise other parameters of the social network simulators, including preferences and the weights of preferences (See Table A).

Table A. The parameters of the DT-CNSs.

Parameters	p	w^p	h	w^h
$DT{-}CNS_U^{P+}$	1	1.00	1	0.00
$DT{-}CNS_U^{P-}$	-1	1.00	1	0.00
$DT{-}CNS_U^{H+}$	1	0.00	1	1.00
$DT{-}CNS_U^{H-}$	1	0.00	-1	1.00
$DT{-}CNS_U^{PH}$	-1	0.05	1	0.08
$DT{-}CNS_B^{P+}$	1	1.00	1	0.00
$DT{-}CNS_B^{P-}$	-1	1.00	1	0.00
$DT{-}CNS_B^{H+}$	1	0.00	1	1.00
$DT{-}CNS_B^{H-}$	1	0.00	-1	1.00
$DT{-}CNS_B^{PH}$	-1	0.03	1	0.06
$DT{-}CNS_I^{P+}$	1	1.00	1	0.00
$DT{-}CNS_I^{P-}$	-1	1.00	1	0.00
$DT{-}CNS_I^{H+}$	1	0.00	1	1.00
$DT{-}CNS_I^{H-}$	1	0.00	-1	1.00
$DT{-}CNS_I^{PH}$	1	0.68	-1	0.73
$DT{-}CNS_L^{P+}$	1	1.00	1	0.00
$DT{-}CNS_L^{P-}$	-1	1.00	1	0.00
$DT{-}CNS_L^{H+}$	1	0.00	1	1.00
$DT{-}CNS_L^{H-}$	1	0.00	-1	1.00
$DT{-}CNS_L^{PH}$	1	0.02	-1	0.08
$DT{-}CNS_R^{P+}$	1	1.00	1	0.00
$DT{-}CNS_R^{P-}$	-1	1.00	1	0.00
$DT{-}CNS_R^{H+}$	1	0.00	1	1.00
$DT{-}CNS_R^{H-}$	1	0.00	-1	1.00
$DT{-}CNS_R^{PH}$	1	0.02	-1	0.06
