# Supplementary material for: Towards Digital Twin-Oriented Complex Networked Systems: Introducing heterogeneous node features and interaction rules
Source: PLoS One. 2024 Jan 2;19(1):e0296426. doi: 10.1371/journal.pone.0296426 (PMC10760715; doi:10.1371/journal.pone.0296426)
Supplement: S2 Appendix — (PDF) [file pone.0296426.s002.pdf]

S2 Appendix.

In our experiments, we employ JS divergence to evaluate the differences between distribution-based network patterns, including degree distributions, clustering coefficient distributions and shortest path length distributions. Tab. A presents the JS divergence values between different network patterns.

Table A. The JS divergence between the network patterns of simulated networks and the target network.

Network Patterns	Degree Distribution	Clustering Coefficient Distribution	Shortest Path Length Distribution
$DT-CNS_U^{P+}$	0.51	0.63	0.29
$DT-CNS_U^{P-}$	0.53	0.68	0.28
$DT-CNS_U^{H+}$	0.36	0.68	0.12
$DT-CNS_U^{H-}$	0.43	0.69	0.35
$DT-CNS_U^{PH}$	0.26	0.60	0.00
$DT-CNS_B^{P+}$	0.50	0.63	0.09
$DT-CNS_B^{P-}$	0.50	0.66	0.16
$DT-CNS_B^{H+}$	0.45	0.55	0.01
$DT-CNS_B^{H-}$	0.50	0.69	0.34
$DT-CNS_B^{PH}$	0.27	0.41	0.01
$DT-CNS_I^{P+}$	0.56	0.68	, 0.31
$DT-CNS_I^{P-}$	0.54	0.68	0.30
$DT-CNS_I^{H+}$	0.46	0.81	0.22
$DT-CNS_I^{H-}$	0.42	0.67	0.41
$DT-CNS_I^{PH}$	0.33	0.70	0.06
$DT-CNS_L^{P+}$	0.56	0.70	0.37
$DT-CNS_L^{P-}$	0.53	0.68	0.01
$DT-CNS_L^{H+}$	0.49	0.53	0.01
$DT-CNS_L^{H-}$	0.53	0.68	0.37
$DT-CNS_L^{PH}$	0.28	0.45	0.01
$DT-CNS_R^{P+}$	0.51	0.69	0.01
$DT-CNS_R^{P-}$	0.59	0.72	0.40
$DT-CNS_R^{H+}$	0.47	0.58	0.01
$DT-CNS_R^{H-}$	0.55	0.68	0.39
$DT-CNS_R^{PH}$	0.28	0.50	0.01
