# Supplementary material for: Towards Digital Twin-Oriented Complex Networked Systems: Introducing heterogeneous node features and interaction rules
Source: PLoS One. 2024 Jan 2;19(1):e0296426. doi: 10.1371/journal.pone.0296426 (PMC10760715; doi:10.1371/journal.pone.0296426)
Supplement: S3 Appendix — (PDF) [file pone.0296426.s003.pdf]

S3 Appendix.

In this study, we select a single seed for epidemic spread in the initial stage based on the largest node degree (See Tab. A).

Table A. The single seed selection based on the largest node degree.

Features	Rules	Seed	
		Node degree	Age group
Uniform	$P+$	70	80-89
	$P-$	68	0-9
	$H+$	52	80-89
	$H-$	43	20-29
	PH	63	0-9
Bell	$P+$	77	70-79
	$P-$	75	10-19
	$H+$	73	70-79
	$H-$	47	40-49
	PH	68	0-9
Inverse bell	$P+$	66	80-89
	$P-$	67	0-9
	$H+$	42	80-89
	$H-$	40	20-29
	PH	63	80-89
Left skewed	$P+$	64	80-89
	$P-$	81	20-29
	$H+$	73	20-29
	$H-$	50	70-79
	PH	67	0-9
Right skewed	$P+$	79	50-59
	$P-$	62	0-9
	$H+$	74	70-79
	$H-$	51	10-19
	PH	74	70-79

As shown in Tab. A, the seeds selected for all the modelling paradigms generally fall in an age group which are preferred by much denser age groups. For example, the old (young) nodes are preferred by all other nodes in $DT{-}CNS^{P+}$ paradigms ($DT{-}CNS^{P-}$ paradigms) and thus have the largest number of connections, which almost directly connect all the connected nodes in the respective model (See Tab. 8 in the manuscript).
