# Supplementary material for: Towards Digital Twin-Oriented Complex Networked Systems: Introducing heterogeneous node features and interaction rules
Source: PLoS One. 2024 Jan 2;19(1):e0296426. doi: 10.1371/journal.pone.0296426 (PMC10760715; doi:10.1371/journal.pone.0296426)
Supplement: S4 Appendix — (PDF) [file pone.0296426.s004.pdf]

S4 Appendix.

In this study, we calculate the infection occurrences within different distances to the seed node (See Fig. A).

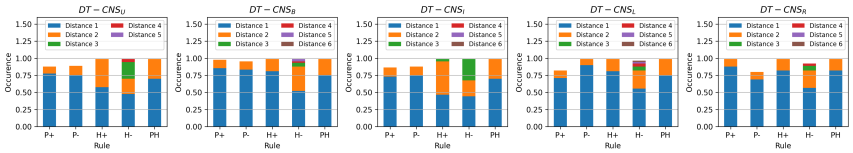

(a) 1.0 transmissibility given an exposure

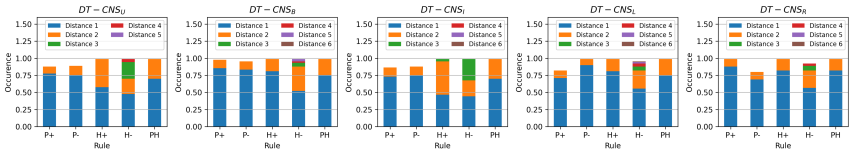

(b) 0.8 transmissibility given an exposure

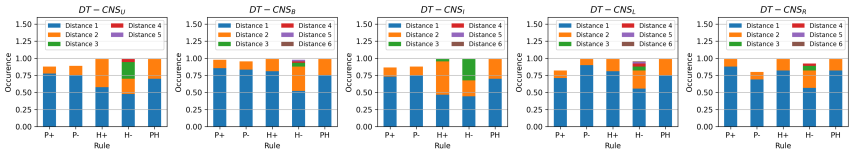

(c) 0.6 transmissibility given an exposure

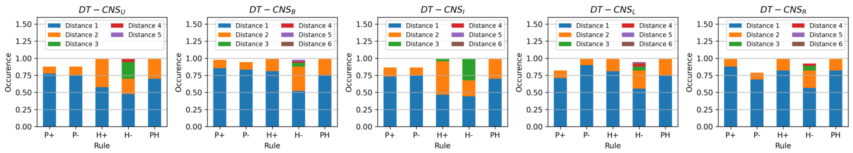

(d) 0.4 transmissibility given an exposure

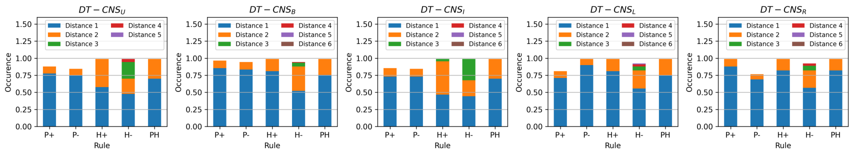

(e) 0.2 transmissibility given an exposure

Fig A. The infection occurrence within certain distance to the seed given different transmissibilities.

In Fig. A, we represent the number of infection occurrences within specific number of steps (edges away) from the first infection. The vertical axis and the horizontal axis each represents the rules of network formation and the infection occurrence among the population (90 nodes in total). As we simulate the epidemic spread for 6 time steps and allow the epidemic propagation by one step (edge) for each time step, nodes within 6 steps away from the first infection are likely to be infected and thus have

infection risks in the epidemic simulations. Nodes out of this range will not be exposed to the infection risks and thus some of the nodes stay uninfected despite the increase of transmissibility. The infection occurrence generally stays the same given any transmissibility between 0.4 and 1.0. Given different rules, the $DT{-}CNS^{P+}$ and the $DT{-}CNS^{P-}$ paradigms can achieve the biggest number of infections within a distance of 1 (one edge away) from the seed; $DT{-}CNS^{H+}$ and $DT{-}CNS^{PH}$ reach the upper limit of 90 within a distance of 2; $DT{-}CNS^{H-}$ gets the biggest number of infections within a distance of around 6. This indicates that the networks, driven by $P+$ or $P-$ rule (preferential attachment to old/young ages), reach the maximum infection occurrence within the first time step as most nodes are directly connected with the seed and can get infected. Given various age distributions, $DT{-}CNS_I$ has the fewest infections within the distance of 1, indicating the less explosive effect of the epidemic outbreak from the seed. The above mentioned phenomenon suggests that the isolation policies imposed on nodes 1 or 2 steps away from the seed can efficiently reduce infection occurrence especially in networks driven by the $H-$ rule, which describes the nodes' preferences for similar features.
