# Supplementary material for: Towards Digital Twin-Oriented Complex Networked Systems: Introducing heterogeneous node features and interaction rules
Source: PLoS One. 2024 Jan 2;19(1):e0296426. doi: 10.1371/journal.pone.0296426 (PMC10760715; doi:10.1371/journal.pone.0296426)
Supplement: S5 Appendix — (PDF) [file pone.0296426.s005.pdf]

S5 Appendix.

To better understand the diversity of age features and their influence on the infection status, we respectively calculate the $PaR(1,1)$ of each age group based on different transmissibilities, age groups and rules (See Fig. A, Fig. B, Fig. C, Fig. D and Fig. E). We also identify the age group where the epidemic starts with a green arrow in the corresponding $PaR(1,1)$ figures.

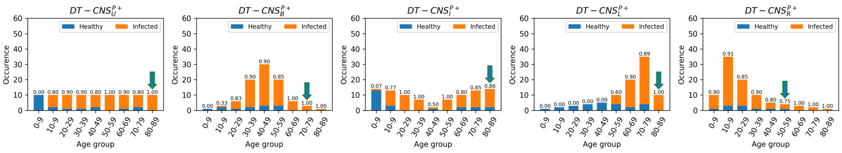

(a) 1.0 transmissibility given an exposure

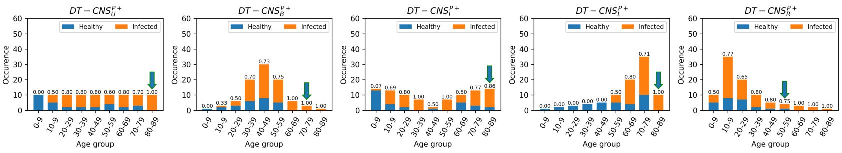

(b) 0.8 transmissibility given an exposure

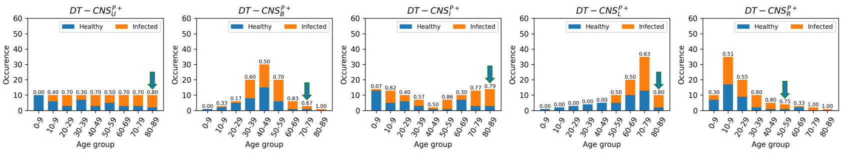

(c) 0.6 transmissibility given an exposure

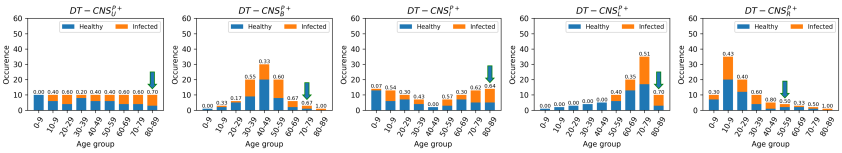

(d) 0.4 transmissibility given an exposure

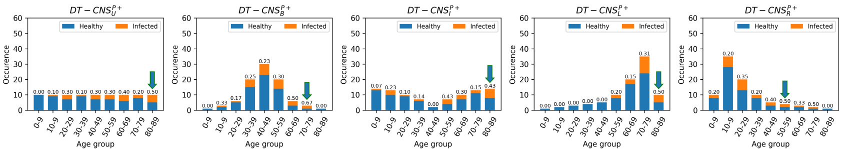

(e) 0.2 transmissibility given an exposure

Fig A. The PaR(1,1) for each age group given different transmissibilities, age distributions and the $P+$ rule.

In Fig. A, the $PaR(1,1)$ increases with the increasing transmissibility. In addition, there is a significant upward trend of $PaR(1,1)$ when the transmissibility changes from 0.2 to 0.4. This indicates an increasing level

of difficulty to resist an epidemic outbreak given an increased transmissibility, because a higher $PaR(1,1)$ value represents a higher proportion of infected nodes and more resources that are needed to keep the epidemic under control. Given the $P+$ rule (preferential attachment to old ages) of network formation but different age distributions, older age groups and the denser age groups, each characterised with higher node degrees and node clustering coefficient, tend to have higher $PaR(1,1)$ values (See Fig. 7, Fig. 8 and Tab. 8 in the manuscript). Due to the features they prefer and the similar preferences within the same age group, these nodes get directly connected with the seed node, exposing the corresponding age group to much higher infection risk. For example, with the $DT{-}CNS_R$ paradigms and the epidemic transmission from older age groups around the age of $50-59$, the younger and denser age groups around the age of $0-39$ have higher $PaR(1,1)$ values due to the significant number of direct connections with the seed node. Therefore, the preference for old ages with the $P+$ rule (positive preferential attachment to age) may pose the old at higher risk. Similarly, the density of age groups can also lead to higher infection risks. However, this is not always the case in reality and is constrained by our assumptions related to features and the corresponding preferences.

As is shown in Fig. B, given $P-$ rule (preferential attachment to young ages) of network formation, young and dense age groups have higher $PaR(1,1)$ values due to the similarly preferred features within the corresponding age group. Most of these nodes get directly connected with the seed node and have higher infection risks. This indicates a relatively lower resistance level to such an epidemic outbreak as a higher proportion of the respective age groups tend to be infected and treated in epidemic control. The higher $PaR(1,1)$ values in young-age groups given the $P+$ rule contrasts with the lower ones given the $P-$ rule (See Fig. A). However, the preferential attachment principles, either $P+$ or $P-$, induces higher infection risks for dense age groups.

As is shown in Fig. C, given the $H+$ rule (preferences for dissimilar ages) of network formation, the age groups, which are denser and have larger age differences with the seed node, have higher $PaR(1,1)$ values due to their similar preferences for dissimilar ages and thus, the significant number of direct connections across the age groups (between the age group of the seed node and the other age groups). Compared with the $DT{-}CNS^{P-}$ models built on negative preferential attachment in age values, the $DT{-}CNS^{H+}$ models have a different seed node but similar $PaR(1,1)$ value distributions in the respective age groups. This is because, in both these cases, the young and dense age groups prefer to be connected with the seed node. Therefore, in the shortest time and distance to the first infection, the differences in $P-$ and $H+$ rules can not lead to significant

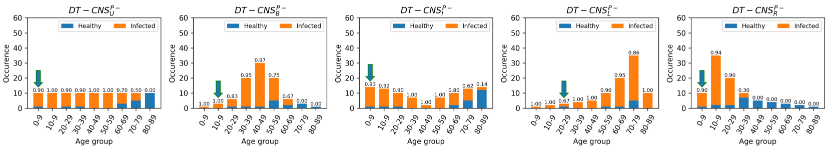

(a) 1.0 transmissibility given an exposure

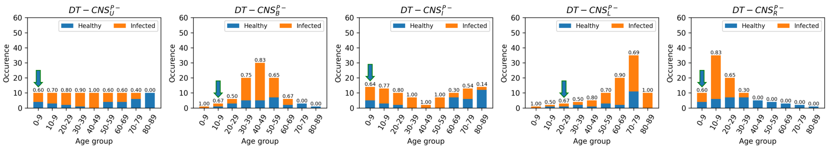

(b) 0.8 transmissibility given an exposure

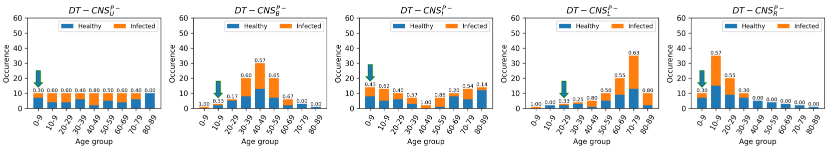

(c) 0.6 transmissibility given an exposure

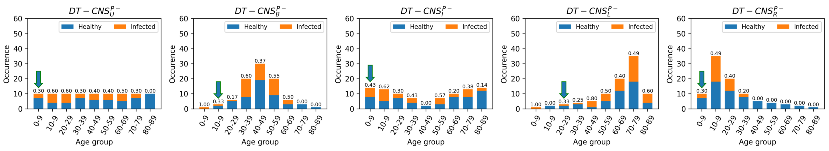

(d) 0.4 transmissibility given an exposure

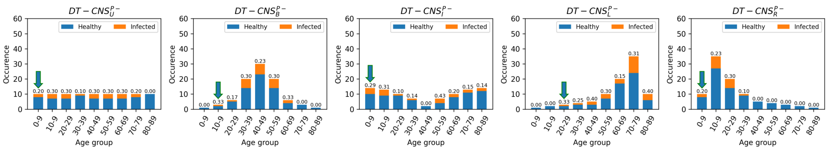

(e) 0.2 transmissibility given an exposure

Fig B. The PaR(1,1) for each age group given different transmissibilities, age distributions and the $P-$ rule.

differences in infection risks. This also indicates that the preference for the seed node for each age group influences their $PaR(1,1)$ values directly.

In Fig. D, given the $H-$ rule (preferences for similar ages) of network formation, fewer age groups get involved in the epidemic spreading process since the homophily effect limits the interactions with the seed node to similar age groups (See Tab. 8 in the manuscript). The $PaR(1,1)$ values are higher for age groups which are dense and in a similar age with the seed node due to the similar preferences for similar features ($H-$ rule). In contrast with the other rules (See Fig.A, Fig. B, Fig. C and Fig. D), the lower infection risk, as indicated by smaller $PaR(1,1)$ values, represents a higher resistance level to the epidemic outbreak in the early stages of epidemics and distance to the first infection.

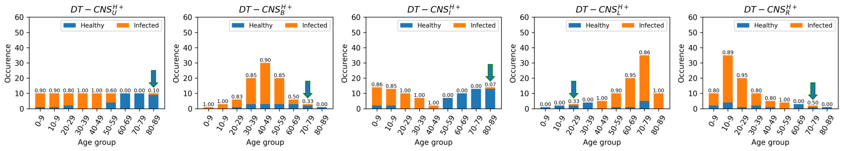

(a) 1.0 transmissibility given an exposure

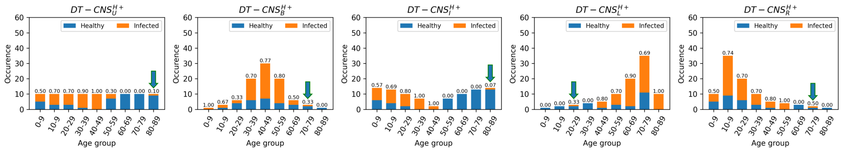

(b) 0.8 transmissibility given an exposure

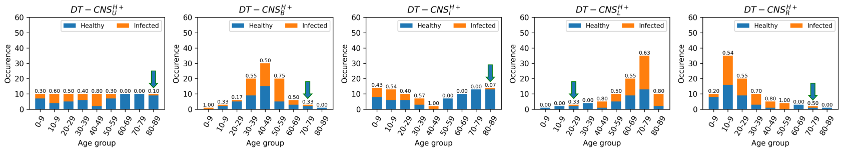

(c) 0.6 transmissibility given an exposure

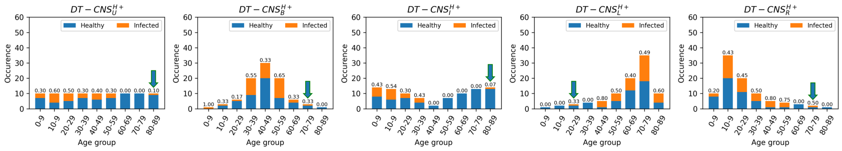

(d) 0.4 transmissibility given an exposure

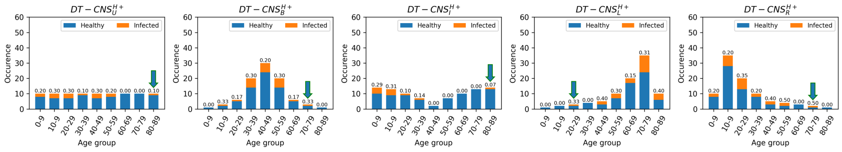

(e) 0.2 transmissibility given an exposure

Fig C. The PaR(1,1) for each age group given different transmissibilities, age distributions and the $H+$ rule.

In Fig. E, the PH rule (optimised preferences for ages and age difference) of network formation optimally combines the effect of preferential attachment and homophily principles to approach the target degree distributions of the scale-free networks. The $DT\!-\!CNS_U^{PH}$ and the $DT\!-\!CNS_B^{PH}$ models combine the negative preferential attachment in ages and the heterophily effect related to the preferences for different ages. For these two models, the old age groups, dissimilar to the seed node and sharing similar preferences for young nodes $(0-9)$, have high $PaR(1,1)$ values. In contrast, the $DT\!-\!CNS_I^{PH}$, $DT\!-\!CNS_L^{PH}$ and the $DT\!-\!CNS_R^{PH}$ models combine the positive preferential attachment in ages and the heterophily effect related to the preferences for different ages. For

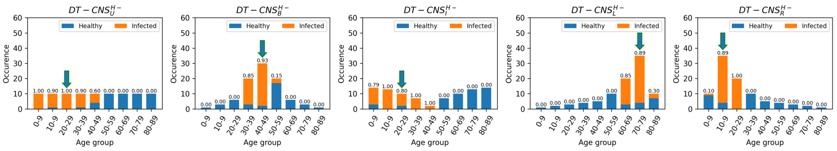

(a) 1.0 transmissibility given an exposure

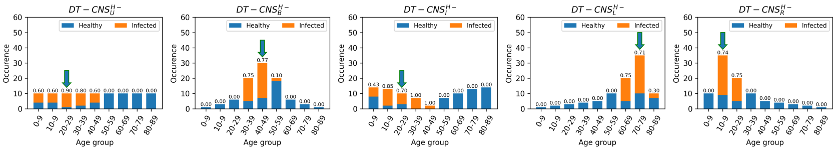

(b) 0.8 transmissibility given an exposure

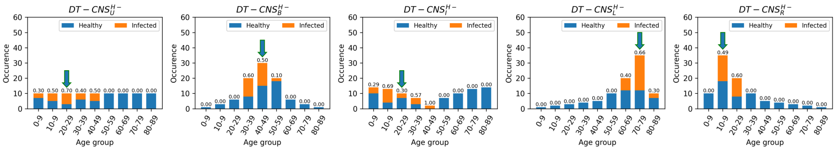

(c) 0.6 transmissibility given an exposure

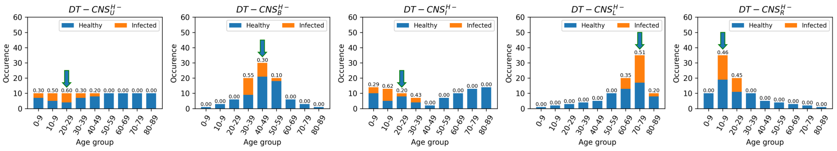

(d) 0.4 transmissibility given an exposure

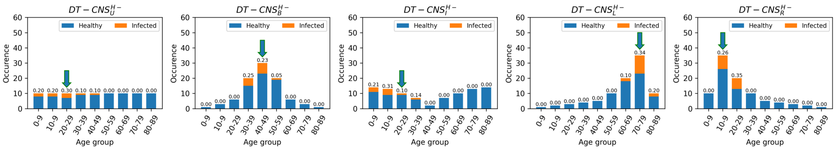

(e) 0.2 transmissibility given an exposure

Fig D. The PaR(1,1) for each age group given different transmissibilities, age distributions and the $H-$ rule.

these models, the age groups, which are younger (younger than 60) and denser, have high $PaR(1,1)$ values. The phenomena mentioned above indicate the influence of density in age groups on the corresponding $PaR(1,1)$ values. Denser age groups, despite the differences and complexities in preferences, generally have higher infection risks and correspondingly lower resistance levels to the epidemic outbreak.

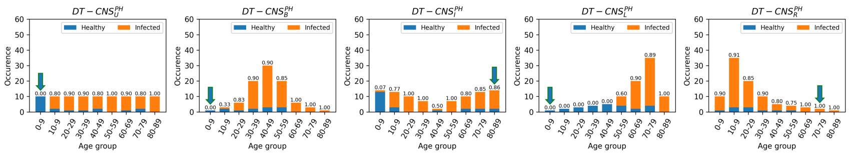

(a) 1.0 transmissibility given an exposure

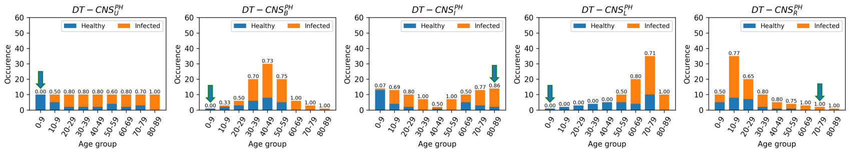

(b) 0.8 transmissibility given an exposure

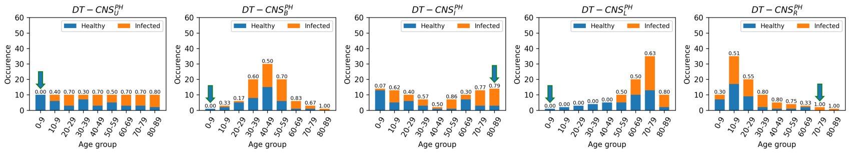

(c) 0.6 transmissibility given an exposure

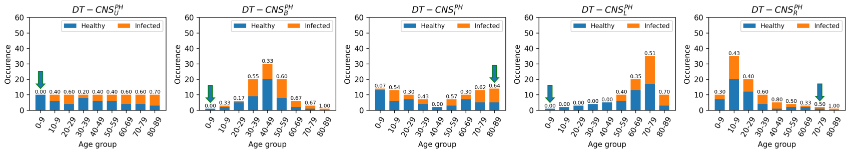

(d) 0.4 transmissibility given an exposure

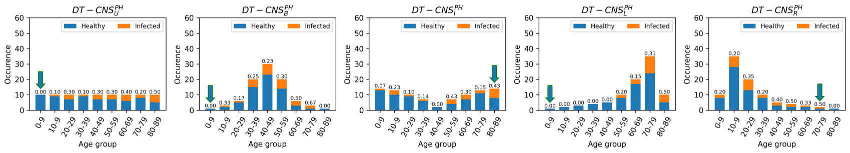

(e) 0.2 transmissibility given an exposure

Fig E. The PaR(1,1) for each age group given different transmissibilities, age distributions and the PH rule.
