# Supplementary figures and images for: Towards Digital Twin-Oriented Complex Networked Systems: Introducing heterogeneous node features and interaction rules
Source: PLoS One. 2024 Jan 2;19(1):e0296426. doi: 10.1371/journal.pone.0296426 (PMC10760715; doi:10.1371/journal.pone.0296426)

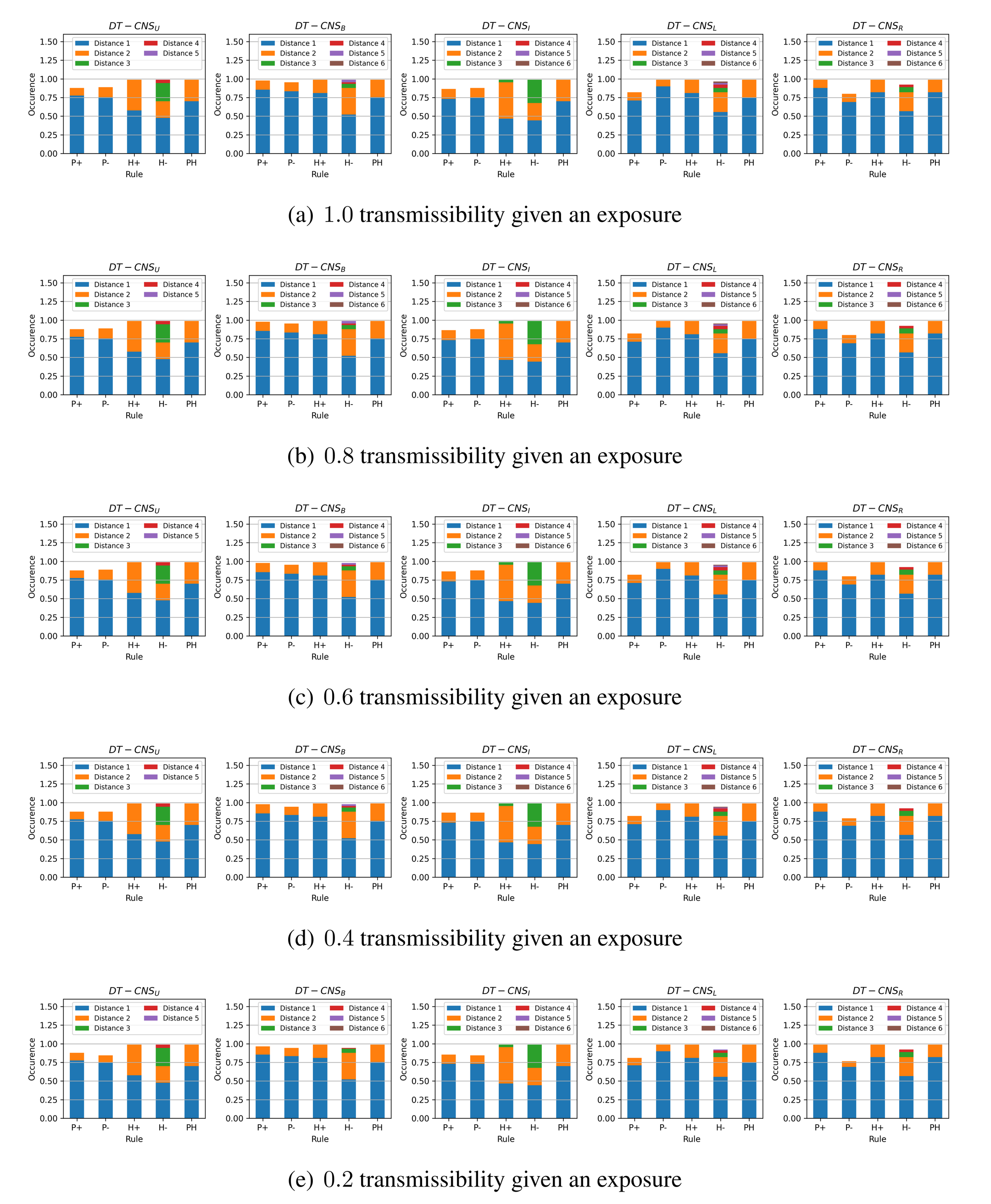

Supplement: S1 Fig — (TIF) [file pone.0296426.s006.tif]

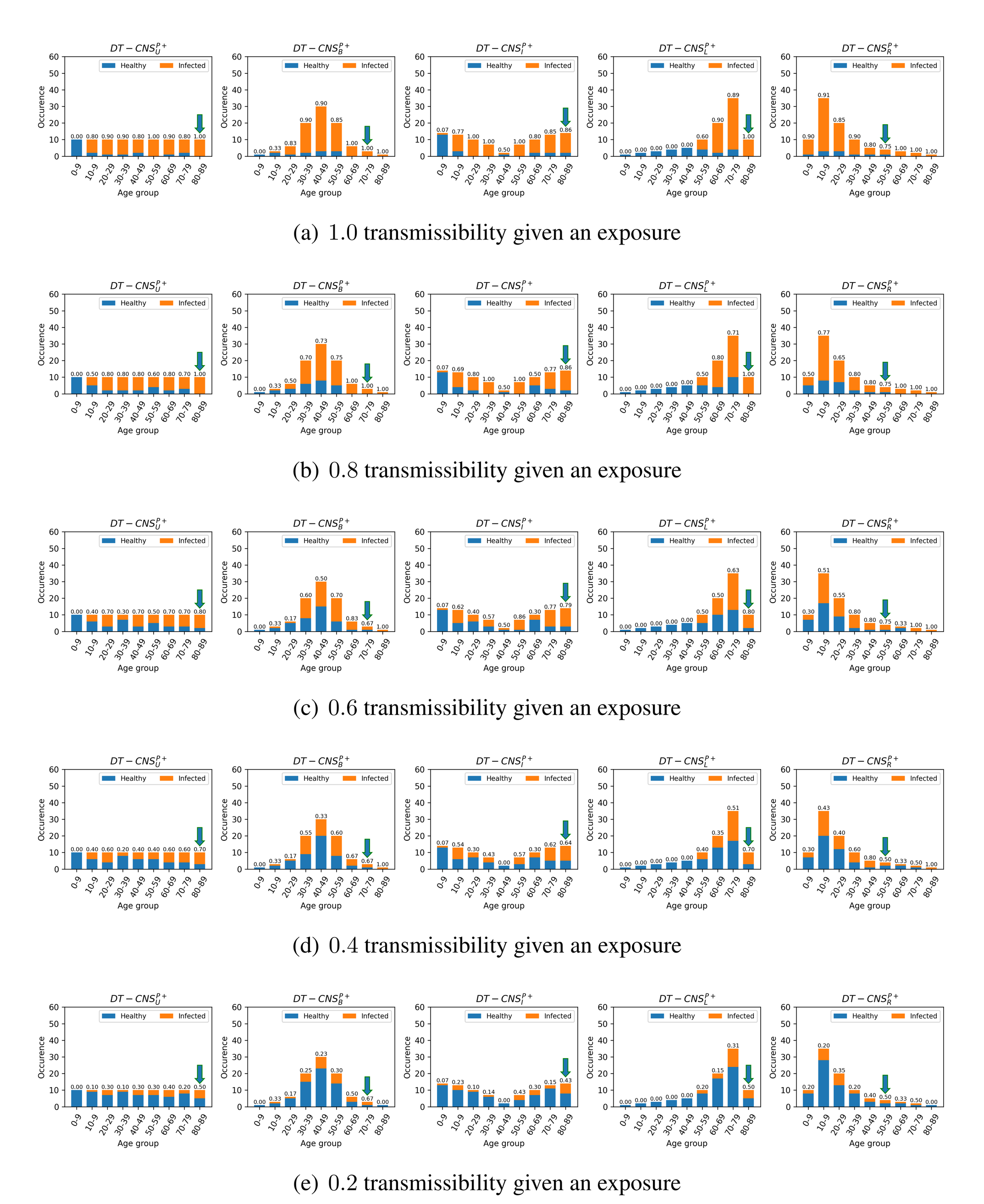

Supplement: S2 Fig — (TIF) [file pone.0296426.s007.tif]

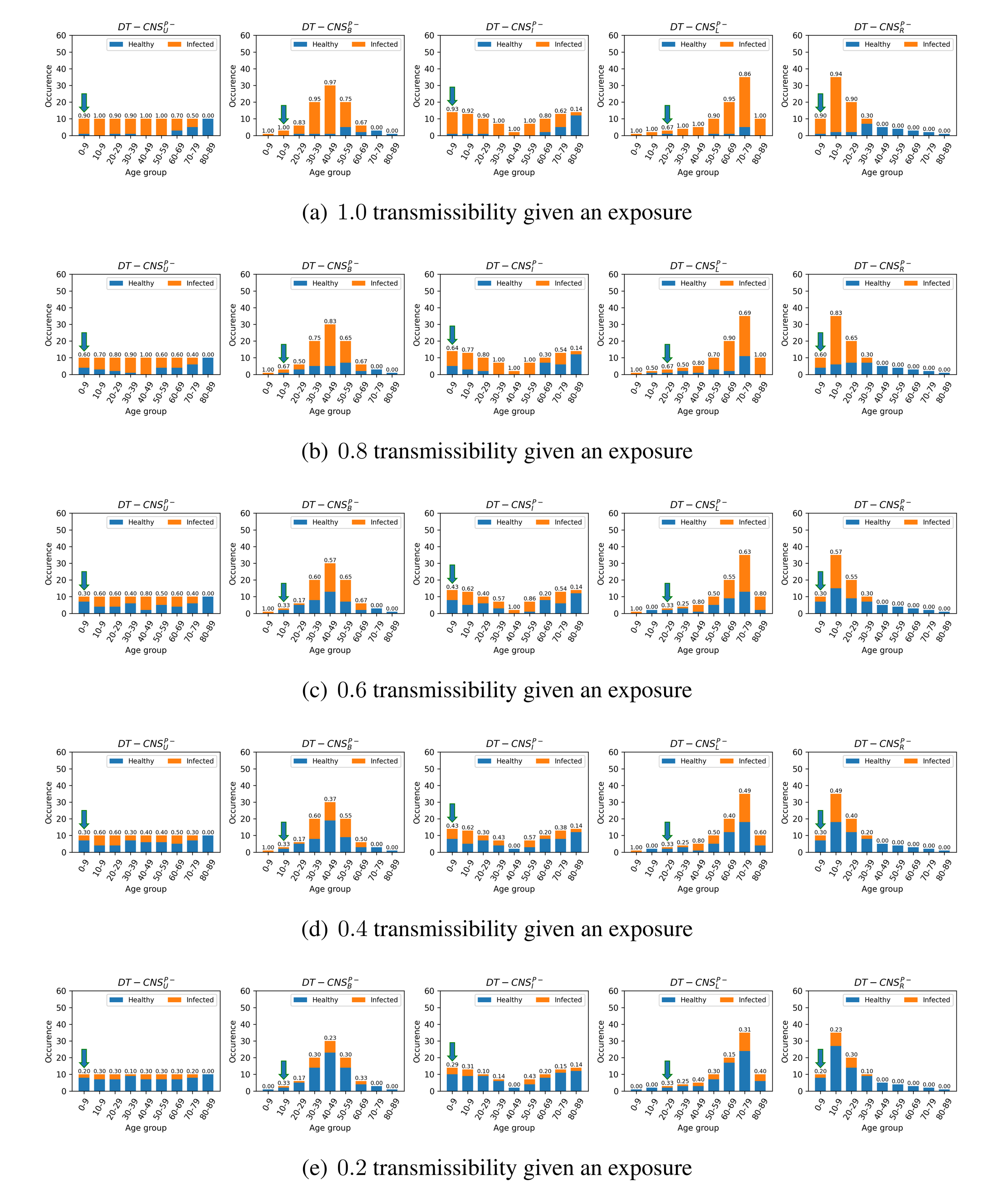

Supplement: S3 Fig — (TIF) [file pone.0296426.s008.tif]

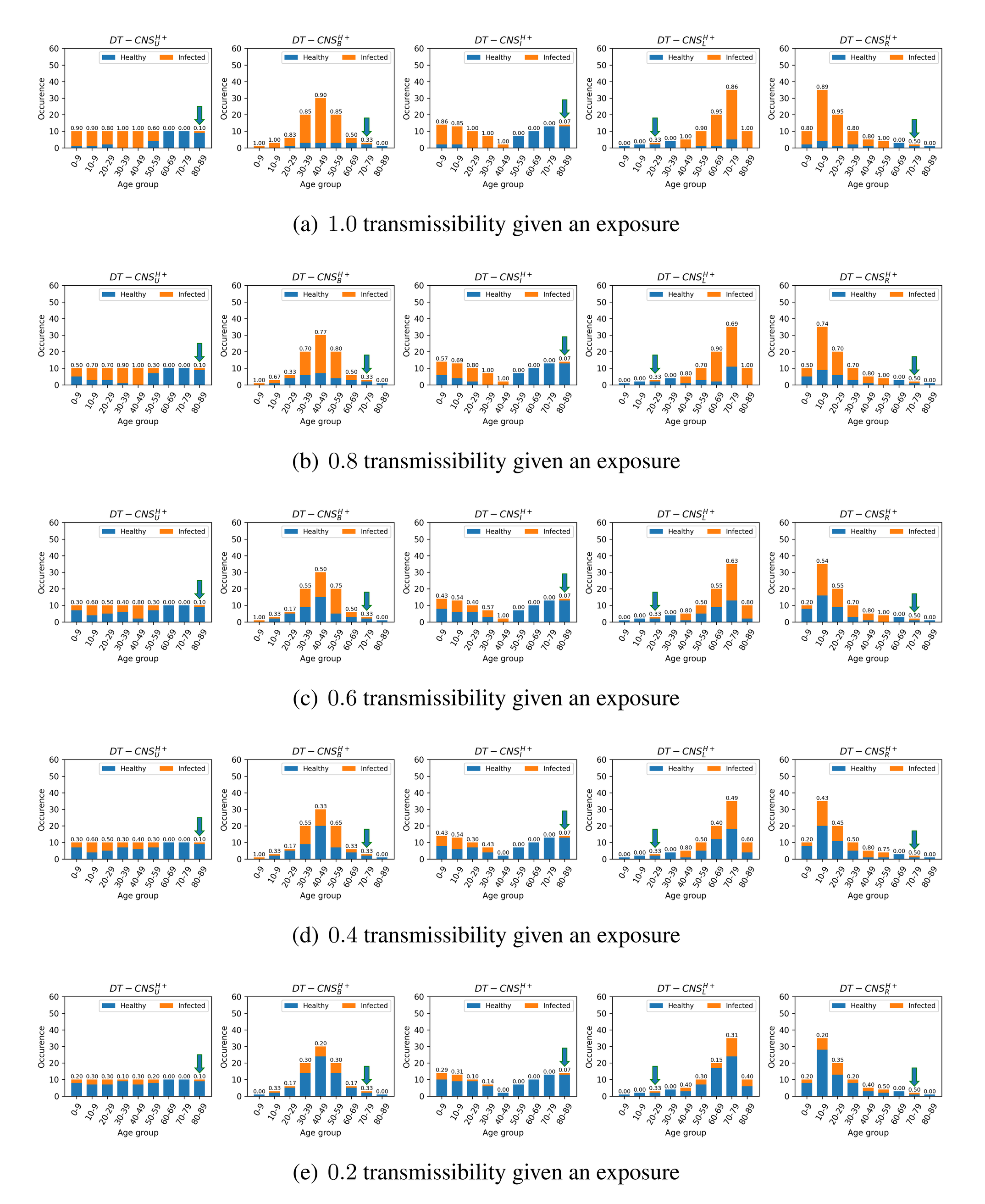

Supplement: S4 Fig — (TIF) [file pone.0296426.s009.tif]

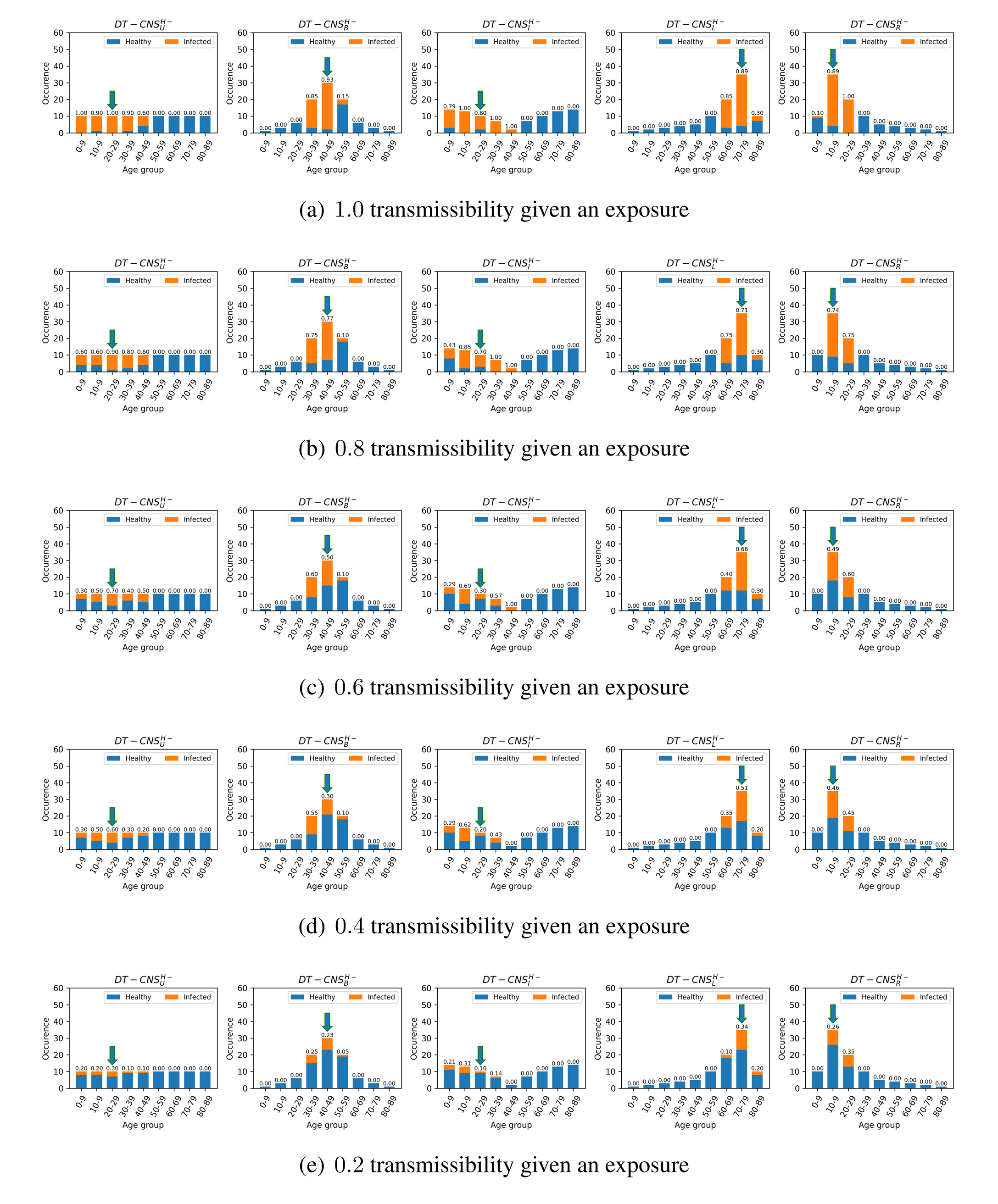

Supplement: S5 Fig — (TIF) [file pone.0296426.s010.tif]

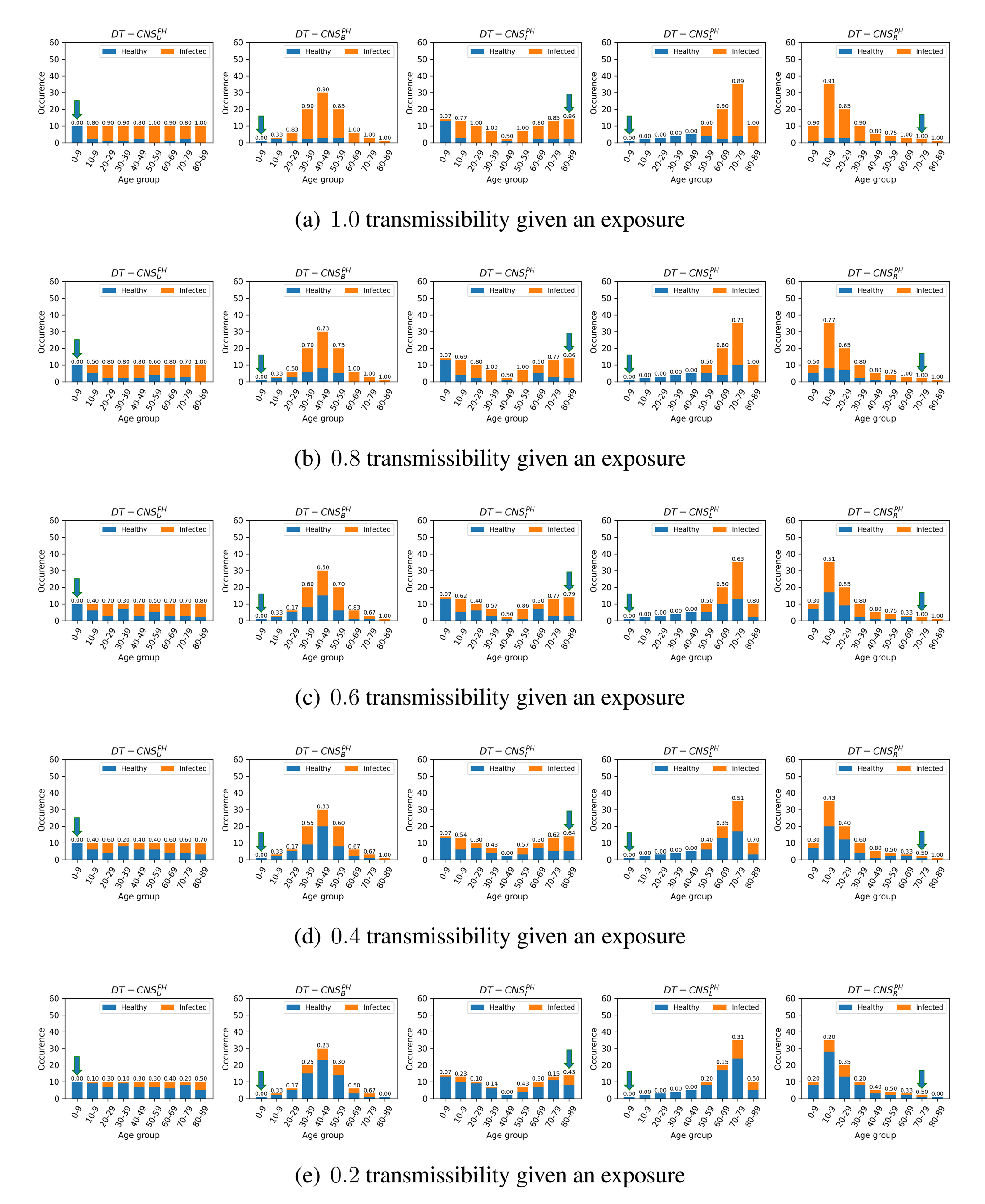

Supplement: S6 Fig — (TIF) [file pone.0296426.s011.tif]
